# Fib-4 score is able to predict intra-hospital mortality in 4 different SARS-COV2 waves

**DOI:** 10.1007/s11739-023-03310-y

**Published:** 2023-07-25

**Authors:** Luca Miele, Marianxhela Dajko, Maria Chiara Savino, Nicola D. Capocchiano, Valentino Calvez, Antonio Liguori, Carlotta Masciocchi, Lorenzo Vetrone, Irene Mignini, Tommaso Schepis, Giuseppe Marrone, Marco Biolato, Alfredo Cesario, Stefano Patarnello, Andrea Damiani, Antonio Grieco, Vincenzo Valentini, Antonio Gasbarrini, Gemelli against COVID Group

**Affiliations:** 1 Dipartimento di Scienze Mediche e Chirurgiche (DiSMeC), Fondazione Policlinico Gemelli IRCCS, Università Cattolica del S. Cuore, 8, Largo Gemelli, 00168 Rome, Italy; 2 Department of Medicina e Chirurgia Traslazionale, Università Cattolica Del Sacro Cuore, Rome, Italy; 3 Department Diagnostica per Immagini, Radioterapia Oncologica ed Ematologia, Fondazione Policlinico Gemelli IRCCS, Rome, Italy; 4 Gemelli Generator Real World Data Unit, Fondazione Policlinico Universitario Agostino Gemelli IRCCS, Rome, Italy; 5 Gemelli Digital Medicine and Health, Fondazione Policlinico Universitario Agostino Gemelli IRCCS, Rome, Italy

**Keywords:** COVID-19, SARS-COV2, Fibrosis, Liver function test, FIB-4

## Abstract

Increased values of the FIB-4 index appear to be associated with poor clinical outcomes in COVID-19 patients. This study aimed to develop and validate predictive mortality models, using data upon admission of hospitalized patients in four COVID-19 waves between March 2020 and January 2022. A single-center cohort study was performed on consecutive adult patients with Covid-19 admitted at the Fondazione Policlinico Gemelli IRCCS (Rome, Italy). Artificial intelligence and big data processing were used to retrieve data. Patients and clinical characteristics of patients with available FIB-4 data derived from the Gemelli Generator Real World Data (G2 RWD) were used to develop predictive mortality models during the four waves of the COVID-19 pandemic. A logistic regression model was applied to the training and test set (75%:25%). The model's performance was assessed by receiver operating characteristic (ROC) curves. A total of 4936 patients were included. Hypertension (38.4%), cancer (12.15%) and diabetes (16.3%) were the most common comorbidities. 23.9% of patients were admitted to ICU, and 12.6% had mechanical ventilation. During the study period, 762 patients (15.4%) died. We developed a multivariable logistic regression model on patient data from all waves, which showed that the FIB-4 score > 2.53 was associated with increased mortality risk (OR = 4.53, 95% CI 2.83–7.25; *p* ≤ 0.001). These data may be useful in the risk stratification at the admission of hospitalized patients with COVID-19.

**Supplementary Information:**

The online version contains supplementary material available at 10.1007/s11739-023-03310-y.

## Introduction

Liver injury is frequent in patients with COVID-19, with a prevalence of up to 70% [1, 2]. Greater liver functional impairment is associated with more severe forms of the disease. Subjects with chronic liver disease have a higher risk of severe clinical events related to SARS-CoV-2 infection [3].

The Fibrosis-4 Index (FIB-4) is a score derived from routine blood tests, including AST, ALT, platelets (PLT), and age. This score predicts mortality and not-liver-related clinical outcome [4, 5]. Some studies have explored the association between COVID-19 outcomes and FIB-4. While some were prognostic models.[6–9], others referred to intensive care admission and the need for mechanical ventilation [7, 10, 11]. Machine-learning approaches are a valuable tool for decision-making and capacity allocation and are also increasingly used for COVID-19 patients [12–16].

We evaluated clinical and laboratory data of all patients with COVID-19 admitted to Fondazione Policlinico Universitario Gemelli IRCCS (Rome, Italy) in terms of in-hospital mortality, mechanical ventilation need, length of hospital stays (LOS) within 10 days and admission to the intensive care unit (ICU). We aimed to develop prognostic models for in-hospital mortality during the first four waves, using a machine-learning approach on routinely collected clinical data of patients with available FIB-4 scores.

## Methods

This retrospective study was approved by the local ethic committee (ID: 3119). It included all patients with a confirmed diagnosis of COVID-19 at Fondazione Policlinico Universitario A Gemelli IRCCS during four waves of the COVID-19 pandemic: (i) First wave: March–June 2020; (ii) Second wave (October 2020 to February 2021); (iii) Third wave (March–June 2021); (iv) Fourth wave (November 2021 to January 2022). Diagnosis of COVID-19 was defined as the presence of ≥ 1 positive RT-PCR SARS-CoV-2 test from a nasopharyngeal swab at admission.

The research was conducted in accordance with both the Declarations of Helsinki and Istanbul, all research was approved by the ethics review committee of Fondazione Policlinico Gemelli IRCCS, Rome, Italy (ID: 3119).

### Outcomes

The primary study outcome was in-hospital mortality. Secondary outcomes were ICU admission, mechanical ventilation use, and the patient was discharged within 10 days from admission.

### Data source

Patients’ data were retrieved from electronic healthcare records using the hospital's data science facility Gemelli Generator Real World Data (G2 RWD), a recently developed data analytics and artificial intelligence platform [12, 17]. All data were deidentified before extraction. The G2 RWD repeatable framework leverages several artificial intelligence (AI) techniques to build the disease-specific data model and data set: in this study, the COVID Data Mart, already described elsewhere [12]. COVID-19 Data Mart [12], is built on standard procedures that apply natural language processing algorithms to medical reports. These procedures are based on sentences/words tokenization and a rule-based approach supported by annotations defined by clinical subject matter experts (SMEs) [12].

For each patient, information related to comorbidities, symptoms, vital signs and laboratory exams, demographic and clinical data prior to therapy for SARS-CoV-2 infection were evaluated (Supplementary material).

The FIB-4 index was calculated using the following formula: age (years) × AST (IU/L)/[platelet count (10^9^/L)/√ALT (IU/L)] [18].

### Data analysis

Data were analysed by descriptive statistics. Univariate analysis was used to compare potential predictors during each COVID-19 wave for in-hospital mortality, mechanical ventilation needs, ICU admission and discharge within 10 days from admission. Data were compared by the *χ*^2^ test, the *T*-tests, or the Mann–Whitney *U* test, as appropriate. A univariate logistic regression model was used to evaluate how the components of the FIB-4 score (age, PLT, ALT and AST) and the score itself influenced the probability of death in each of the four waves.

Furthermore, a multivariate logistic regression model focusing specifically on FIB-4 was fitted on patients' data for each wave. For the statistical modeling, the whole dataset was randomly split into two parts: the training set (75% of the total observations) and the test set (25% of the total observations). A stratified sampling strategy was adopted to preserve patient distributions across waves (Supplementary material).

The algorithm led to the following discretization: NLR Score was grouped into three groups (< 3.87, 3.87–7.51 and > 7.51), FIB-4 score was grouped into two groups (< 2.53 and ≥ 2.53), hemoglobin was grouped into two groups (< 12.9 g/dL and ≥ 12.9 g/dL), hematocrit was grouped into two groups (< 38.30% and ≥ 38.30%), calcium was grouped into three groups (< 8.94 mEq/L, 8.94–9.40 mEq/L and > 9.40 mEq/L), urea nitrogen was grouped into three groups (< 17.00, 17.00–26.00, > 26.00 mg/dL) and Charlson score was grouped into four groups (< 3.00, 3.00–4.00, 4.00–6.00, > 6.00).

A logistic regression model was trained based on the training set using forward features selection to remove any features that did not significantly affect survival prediction.

A new logistic regression model that included only the significant variables coming from forward features selection and FIB-4 score was estimated to reduce model dimensionality. Moreover, a 'wave' variable was included in the final model representing differences across waves in clinical characteristics and organizational factors.

The performance of the final logistic regression model was evaluated on the test set through a receiver operating characteristic (ROC) curve analysis and the resulting AUC. The logistic regression model was optimized to maximize the Youden index. The performance of the model after tuning is shown in a confusion matrix and by computing accuracy, sensitivity, specificity, and negative predictive value.

To further investigate the FIB-4 score discriminative power on in-hospital mortality, the Kaplan–Meier method was used; results were compared by log-rank test.*p* ≤ 0.05 was considered significant unless otherwise stated. All statistical analyses were performed using R software, version 4.0.3 (R Foundation for Statistical Computing, Vienna, Austria).

## Results

### Cohort characteristics

A total of 4936 patients were evaluated. Demographic data, comorbidities, clinical data at admission, and clinical outcomes are shown in Supplementary Table 1.

Briefly, over the entire timeframe, 1981 (40.2%) patients were discharged within 10 days from admission, and 762 (15.4%) did not survive. The median LOS was 11 days [IQR 6–20 days]. The frequency of ICU admission and mechanical ventilation was 23.9% (*n* = 1178) and 12.6% (*n* = 624), respectively.

**Fig. 1:**
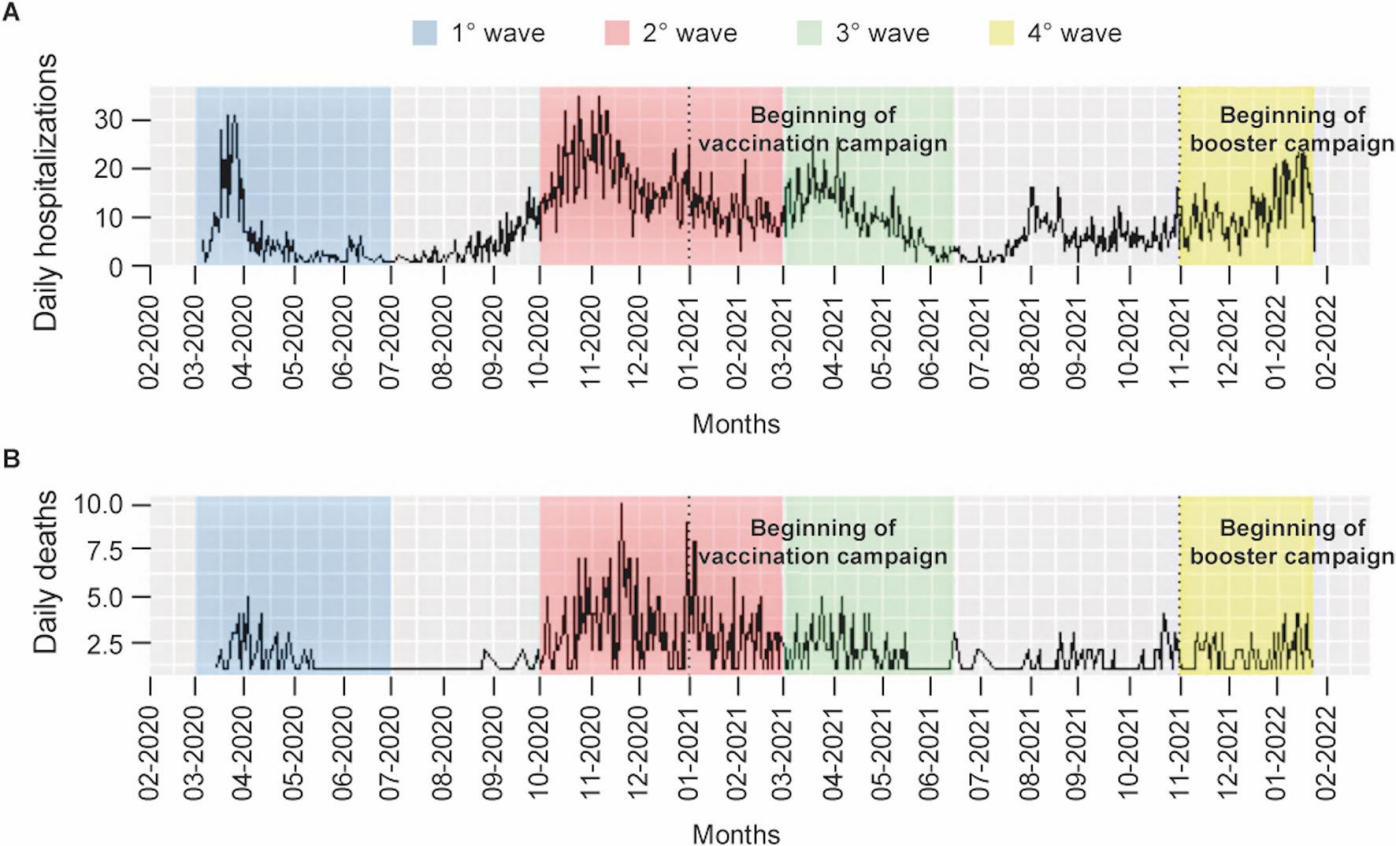
**A** Daily hospital admissions and **B** daily deaths at Fondazione Policlinico Universitario A. Gemelli IRCCS across COVID waves

Daily hospital admissions and deaths across four COVID-19 waves are displayed in Fig. 1, while each wave’s demographic characteristics and outcomes distribution are depicted in Supplementary Table 2. During the second wave, which covered a longer period than the others, in-hospital mortality was higher than in the other waves (448/2336, 19% vs 85/570, 15% in the second wave vs 133/1064, 12% in the third one and vs 96/966, 10% in the fourth wave; *p* < 0.001). The beginning of the vaccination campaign in 2021 led to a decrease in daily counts of admissions and deaths. Discharge within 10 days shows a positive trend, with an increasing percentage of patients discharged with a shorter length of stay (26%, 39%, 42%, and 48% from the first to the fourth wave, respectively).

FIB-4 score did not differ across waves.

### In-hospital mortality: primary outcome

The median age of patients who survived was 64 years [IQR 53–76 years], while the median age of patients who did not survive was 83 years [IQR 78–88 years], *p* < 0.01 during the first wave (Table 1).

**Table 1:** In hospital mortality in the four pandemic waves

	Survivors	Non-survivors	*p*-values	Survivors	Non-survivors	*p*-values	Survivors	Non-survivors	*p*-values	Survivors	Non-survivors	*p*-values
Demographics												
No. patients	484	85		1888	448		931	133		870	96	
Age (years)	64 (53–76)	83 (78–88)	< 0.01	65 (53–77)	80 (71–86)	< 0.01	60 (49–73)	76 (69–83)	< 0.01	62 (47–75)	81 (68–86)	< 0.01
Male	291 (60)	49 (57.6)	0.72	997 (52.8)	272 (60.7)	< 0.01	515 (55.3)	85 (63.9)	0.06	442 (50.0)	56 (58.3)	0.16
BMI (kg/m^2^)	25.3 (23.4–27.6)	24.4 (21.4–27.5)	0.1	26.0 (23.8–29.2)	25.7 (23.3–28.4)	0.02	26.5 (23.9–30.1)	27.3 (24.2–30.8)	0.61	26.3 (23.4–30.0)	26.8 (23.6–31.2)	0.23
Comorbidities												
Obesity (BMI > 30 kg/m^2^)	62 (14.2)	10 (15.1)	0.85	314 (21.5)	55 (18.4)	0.24	216 (25.8)	30 (27.5)	0.73	167 (25.4)	26 (33.3)	0.14
Diabetes	59 (12.19)	19 (22.35)	0.02	325 (17.47)	102 (22.77)	0.01	120 (13.03)	20 (15.04)	0.5	127 (14.6)	28 (29.17)	< 0.01
Hypertension	163 (33.68)	39 (45.88)	0.04	752 (40.43)	212 (47.32)	0.01	318 (34.53)	60 (45.11)	0.02	293 (33.68)	41 (42.71)	0.09
Malignancy	47 (9.71)	15 (17.65)	0.04	216 (11.61)	73 (16.29)	0.01	80 (8.69)	14 (10.53)	0.51	130 (14.94)	20 (20.83)	0.14
Neurological disease	25 (5.17)	15 (17.65)	< 0.01	70 (3.76)	77 (17.19)	< 0.01	21 (2.28)	14 (10.53)	< 0.01	39 (4.48)	6 (6.25)	0.44
Stroke	15 (3.09)	6 (7.06)	0.11	87 (4.61)	43 (9.6)	< 0.01	26 (2.79)	7 (5.26)	0.17	19 (2.18)	6 (6.25)	0.03
Cardiovascular disease	20 (4.13)	13 (15.29)	< 0.01	112 (6.02)	70 (15.62)	< 0.01	46 (4.99)	16 (12.03)	< 0.01	55 (6.32)	9 (9.38)	0.28
Pneumopathy	51 (10.54)	21 (24.71)	< 0.01	156 (8.39)	85 (18.97)	< 0.01	68 (7.38)	19 (14.29)	0.01	63 (7.24)	22 (22.92)	< 0.01
Gastrointestinal disease	7 (1.45)	5 (5.88)	0.02	27 (1.45)	16 (3.57)	0.01	18 (1.95)	5 (3.76)	0.2	24 (2.76)	6 (6.25)	0.11
Chronic kidney disease	20 (4.13)	6 (7.06)	0.26	95 (5.11)	66 (14.73)	< 0.01	28 (3.04)	13 (9.77)	< 0.01	72 (8.28)	19 (19.79)	< 0.01
Chronic liver disease	7 (1.45)	1 (1.18)	1	16 (0.86)	6 (1.34)	0.41	11 (1.19)	1 (0.75)	1	11 (1.26)	1 (1.04)	1
Cirrhosis	1 (0.21)	0 (0)	1	17 (0.91)	8 (1.79)	0.13	4 (0.43)	3 (2.26)	0.05	22 (2.53)	0 (0)	0.16
Thrombosis/pulmonary embolism	20 (4.12)	3 (3.53)	1	38 (2.01)	10 (2.23)	0.71	27 (2.9)	4 (3.01)	1	11 (1.26)	4 (4.17)	0.05
Immunodeficiency	10 (2.07)	1 (1.18)	1	37 (1.99)	15 (3.35)	0.11	19 (2.06)	2 (1.5)	1	37 (4.25)	5 (5.21)	0.6
Symptoms at admission												
Fever	215 (77.06)	42 (76.36)	1	658 (58.59)	129 (62.02)	0.4	467 (67.78)	51 (53.68)	0.01	279 (53.35)	30 (58.82)	0.47
Anosmia/dysgeusia	14 (5.56)	1 (1.89)	0.48	62 (6.55)	1 (0.57)	< 0.01	22 (3.43)	2(2.17)	0.76	6 (1.32)	1 (2.33)	0.47
Cough	145 (53.9)	18 (31.03)	< 0.01	322 (30.84)	50 (26.18)	0.23	213 (32.13)	18 (19.15)	0.01	154 (31.62)	11 (25)	0.4
Dyspnea	144 (52.17)	36 (64.29)	0.11	515 (48.82)	160 (74.77)	< 0.01	386 (57.96)	68 (68.69)	0.05	231 (45.56)	36 (70.59)	< 0.01
Myalgia/arthralgia	52 (20.16)	6 (10.91)	0.13	183 (18.12)	25 (13.37)	0.14	142 (21.26)	10 (10.64)	0.02	80 (16.63)	5 (11.36)	0.52
Gastrointestinal	40 (15.04)	3 (5.56)	0.08	88 (9.07)	10 (5.65)	0.15	53 (8.17)	4 (4.4)	0.29	28 (6.02)	2 (4.55)	1
Hospital admission data												
Oxygen saturation (%)	96 (94–98)	95 (93–97)	0.1	96 (94–97)	95 (92–96)	< 0.01	96(94–97)	95 (93–97)	< 0.01	96 (95–98)	96 (94–97)	0.11
Body temperature (°C)	37.1 (36.1–38.0)	36.9 (36.0–38.0)	0.45	36.2 (36.0–37.2)	36.3 (36.0–37.5)	0.59	36.3 (36.0–37.5)	36.4 (36.0–37.4)	0.63	36.3 (36.0–37.3)	36.3 (36.0–37.4)	0.93
Heart rate (bpm)	80 (70–89)	80 (7–90)	0.36	80 (72–89)	80 (71–91)	0.45	80 (73–90)	80 (70–94)	0.77	80 (73–88)	82 (71–88)	0.47
Respiratory (rate per minute)	13 (12–14)	15 (12–16)	< 0.01	13 (12–14)	13 (12–15)	0.26	14 (12–16)	15 (13–20)	< 0.01	14 (13–15)	14 (13–16)	0.08
Mean blood pressure (mmHg)	90 (83–97)	87 (77–97)	0.14	90 (83–99)	88 (79–99)	< 0.01	93 (85–100)	93 (81–103)	0.99	91 (83–100)	92 (85–107)	0.55
P/F	316.90 206.02–378.22)	230.20 (156.12–351.47)	< 0.01	234.50 (158.27–319.90)	159.80 (106.60–244.90)	< 0.01	210.70 (149.85–300.00)	140.00 (101.80–206.30)	< 0.01	208.40 (147.20–300.00)	137.00 (100.30–218.00)	< 0.01
Laboratory												
Hemoglobin (g/dL)	13.80 (12.30–14.90)	12.90 (10.90–14.30)	< 0.01	13.30 (11.80–14.60)	12.30 (10.60–14.10)	< 0.01	13.60 (12.20–14.90)	12.80 (11.40–14.10)	< 0.01	13.20 (11.55–14.60)	12.70 (11.15–13.80)	0.01
Haematocrit (%)	40.60 (36.90–43.60)	38.80 (33.60–42.50)	0.01	39.20 (35.20–42.80)	37.20 (31.80–41.85)	< 0.01	39.50 (35.90–43.30)	38.40 (34.90–41.90)	0.02	38.00 (33.77–41.42)	37.10 (32.60–39.90)	0.06
Platelets (cell × 10^9^/L)	210.00 (166.00–265.00)	192.00 (139.00–279.00)	0.18	225.00 (174.00–287.25)	201.50 (148.25–261.00)	< 0.01	203.00 (160.00–266.00)	169.00 (134.00–244.00)	< 0.01	208.00 (156.00–270.00)	192.50 (137.00–273.50)	0.14
WBC (cell × 10^9^/L)	6.12 (4.82–8.29)	8.27 (5.57–11.40)	< 0.01	8.01 (5.83–10.93)	8.61 (5.91–11.83)	0.04	7.19 (5.24–9.84)	7.99 (6.04–12.29)	< 0.01	7.43 (5.52–10.78)	8.73 (5.80–13.56)	0.04
Neutrophils (cell × 10^9^/L)	4.50 (3.27–6.30)	6.85 (4.51–9.36)	< 0.01	6.16 (4.21–9.00)	7.01 (4.55–9.94)	< 0.01	5.50 (3.82–8.07)	6.59 (4.53–9.70)	< 0.01	5.60 (3.94–8.31)	7.09 (4.46–11.59)	< 0.01
Lymphocytes (cell × 10^9^/L)	1.14 (0.81–1.51)	0.91 (0.66–1.25)	< 0.01	1.12 (0.78–1.59)	0.89 (0.61–1.34)	< 0.01	0.98 (0.70–1.40)	0.74 (0.52–1.06)	< 0.01	1.02 (0.72–1.55)	0.93 (0.61–1.25)	0.01
D-dimer (ng/mL)	907.00 (472.00–1726.00)	1953.00 (976.50–4045.00)	< 0.01	818.00 (446.00–1757.00)	1601.00 (780.50–3618.50)	< 0.01	690.00 (415.00–1361.00)	1690.00 (778.00–3528.00)	< 0.01	910.00 (506.00–2088.50)	1983.50 (991.00–5110.00)	< 0.01
Procalcitonin (ng/mL)	0.11 (0.07–0.21)	0.23 (0.12–0.60)	< 0.01	0.11 (0.07–0.24)	0.21 (0.10–0.63)	< 0.01	0.10 (0.07–0.19)	0.17 (0.09–0.48)	< 0.01	0.13 (0.08–0.36)	0.33 (0.10–0.74)	< 0.01
IL-6 (ng/L)	14.60 (7.85–29.45)	30.20 (15.72–60.75)	0.01	18.00 (8.50–39.10)	39.00 (16.00–76.00)	< 0.01	19.40 (8.35–37.25)	35.30 (14.45–78.40)	< 0.01	22.50 (10.00–52.20)	38.55 (25.55–74.85)	< 0.01
HS troponin (ng/L)	10.00 (5.50–25.50)	48.00 (20.50–112.00)	< 0.01	11.00 (6.00–27.25)	40.00 (16.00–152.00)	< 0.01	9.00 (5.00–21.00)	29.00 (13.00–86.50)	< 0.01	11.00 (5.00–29.00)	71.50 (20.00–167.25)	< 0.01
Lactate (mmol/L)	1.00 (0.71–1.40)	1.70 (1.35–2.45)	< 0.01	1.00 (0.80–1.50)	1.30 (1.10–2.10)	< 0.01	1.00 (0.70–1.37)	1.30 (1.00–1.90)	< 0.01	1.00 (0.70–1.30)	1.15 (0.90–1.60)	0.01
Lactic dehydrogenase (IU/L)	281.00 (217.00–392.00)	413.00 (310.25–621.75)	< 0.01	294.00 (221.00–402.00)	386.00 (272.00–530.75)	< 0.01	318.00 (238.00–431.00)	424.00 (315.00–557.50)	< 0.01	294.00 (215.00–413.00)	405.00 (306.00–576.50)	< 0.01
Glucose (mg/dL)	108.00 (95.00–123.00)	124.00 (103.00–147.00)	< 0.01	114.00 (95.00–141.00)	128.00 (107.00–166.00)	< 0.01	114.00 (96.00–137.75)	127.50 (107.00–172.50)	< 0.01	111.00 (96.00–136.00)	132.00 (112.00–223.00)	< 0.01
Creatinine (mg/dL)	0.88 (0.72–1.08)	1.26 (0.77–1.74)	< 0.01	0.85 (0.68–1.08)	1.12 (0.82–1.77)	< 0.01	0.81 (0.66–0.98)	1.01 (0.80–1.48)	< 0.01	0.86 (0.69–1.13)	1.23 (0.96–2.36)	< 0.01
ALT (IU/L)	25.00 (15.00–39.00)	23.50 (14.00–39.25)	0.81	23.00 14.00–38.00)	23.00 (14.00–40.00)	0.74	27.00 (17.75–44.00)	27.00 (17.00–41.00)	0.64	22.00 (14.00–39.00)	24.00 (13.00–38.00)	0.89
AST (IU/L)	31.50 (23.25–50.75)	42.50 (28.25–77.00)	0.03	28.00 (20.00–43.25)	42.00 (27.00–66.50)	< 0.01	32.00 (21.75–49.00)	43.00 (22.00–83.00)	0.28	30.00 21.00–48.00)	35.00 (28.50–53.50)	0.05
GGT (IU/L)	32.00 (19.00–62.00)	37.00 (21.00–56.00)	0.62	32.00 (20.00–60.00)	40.00 (20.75–71.00)	0.01	35.00 (22.00–63.00)	34.50 (21.75–71.00)	0.75	31.00 (19.00–60.00)	39.00 (21.25–85.25)	0.08
ALP (IU/L)	67.00 (52.00–86.00)	73.50 (56.50–89.00)	0.2	65.00 (51.00–85.00)	73.00 (55.00–99.00)	< 0.01	58.00 (47.00–78.00)	64.00 (47.75–94.00)	0.04	64.50 (51.00–96.00)	72.50 (53.25–104.50)	0.14
Total bilirubin (mg/dL)	0.60 (0.40–0.80)	0.60 (0.40–0.90)	0.97	0.60 (0.50–0.80)	0.60 (0.40–0.90)	0.62	0.60 (0.50–0.80)	0.60 (0.40–0.90)	0.83	0.60 (0.50–0.80)	0.60 (0.40–0.80)	0.1
Direct bilirubin (mg/dL)	0.40 (0.30–0.50)	0.90 (0.50–1.35)	0.04	0.60 (0.50–0.80)	0.90 (0.60–1.55)	< 0.01	0.50 (0.40–0.70)	1.00 (0.72–1.35)	0.01	0.60 (0.40–1.85)	0.85 (0.78–1.47)	0.53
Albumin (g/L)	35.00 (31.00–38.00)	29.00 (26.00–32.00)	< 0.01	32.00 (29.00–35.00)	29.00 (25.00–32.00)	< 0.01	33.00 (30.00–35.00)	29.00 (25.75–32.00)	< 0.01	32.00 (28.00–36.00)	27.00 (25.00–31.00)	< 0.01
Total cholesterol (mg/dL)	136.00 (116.00–167.00)	110.50 (88.75–131.75)	< 0.01	145.00 (120.00–170.00)	128.00 (106.00–150.50)	< 0.01	139.00 (117.00–165.00)	128.00 (112.75–148.25)	0.01	139.00 (116.00–165.00)	114.50 (99.75–141.00)	< 0.01
HDL cholesterol (mg/dL)	31.00 (25.75–41.00)	24.00 (21.00–33.00)	0.03	35.00 (28.00–42.00)	29.00 (21.00–38.75)	< 0.01	33.00 (27.00–40.00)	30.00 (21.00–42.75)	0.07	35.00 (27.25–43.00)	27.00 (21.00–35.00)	< 0.01
Triglycerides (mg/dL)	110.00 (88.50–157.00)	103.00 (89.25–187.75)	0.77	118.00 (92.00–155.00)	133.00 (98.00–187.75)	< 0.01	117.00 (91.00–150.00)	132.00 (100.75–181.00)	0.01	110.00 (87.00–149.50)	129.00 (92.00–196.00)	0.03
INR	1.04 (1.00–1.09)	1.09 (1.03–1.18)	< 0.01	1.03 (0.97–1.09)	1.08 (1.01–1.18)	< 0.01	1.07 (1.02–1.13)	1.11 (1.06–1.20)	< 0.01	1.02 (0.97–1.08)	1.09 (1.00–1.23)	< 0.01
Noninvasive tests												
FIB-4	1.79 (1.17–2.78)	4.58 (2.40–7.06)	< 0.01	1.59 (0.92–2.76)	3.46 (1.94–5.95)	< 0.01	1.79 (1.03–3.07)	3.96 (2.22–6.33)	< 0.01	1.90 (1.19–3.32)	3.85 (2.50–4.79)	< 0.01
Clinical outcomes												
Length of stay (days)	15 (9.0–25.0)	9 (5.0–15.0)	< 0.01	11 (6.0–19.0)	13 (6.7–23.0)	0.01	11 (6.0–18.0)	14 (8.0–24.0)	< 0.01	10 (5.0–18.0)	12 (7.5–20.0)	0.05
Discharge within 10 days	150 (30.93)	0 (0.00)	< 0.01	916 (48.52)	0 (0)	< 0.01	449 (48.44)	0 (0.00)	< 0.01	466 (53.56)	0 (0.00)	< 0.01
ICU admission	78 (16.08)	37 (43.53)	< 0.01	389 (20.6)	199 (44.42)	< 0.01	201 (21.59)	81 (60.9)	< 0.01	142 (16.32)	51 (53.12)	< 0.01
Mechanical ventilation	52 (10.72)	30 (35.29)	< 0.01	136 (7.2)	145 (32.37)	< 0.01	87 (9.34)	71 (53.38)	< 0.01	65 (7.47)	38 (39.58)	< 0.01

Values shown are the median (IQR) for quantitative variables and number (percentage) for categorical variables. Quantitative variables were compared through *T*-tests or Mann–Whitney *U* test according to their distributions. Categorical variables were compared by *χ*^2^ test. Significance level ≤ 0.05

Data related to ALT were available in 4681, with a median value of 24 IU/L [IQR 15–40 IU/L], and those on AST were available in 1277 patients, with a median of 31 IU/L [IQR 22–49 IU/L]. There were no differences between patients regarding in-hospital mortality status for ALT for all four waves, while AST higher values were associated with mortality for the first and second waves. Higher values of neutrophils, white blood cells (WBC) (*p* < 0.01), procalcitonin, IL-6, D-dimer, glucose, direct bilirubin, INR and LDH were reported in the group of non-survivors compared with survivors (*p* < 0.01 for all comparisons).

PLT count was recorded in 4801 patients, with a median value of 211 10^3^/mm^3^ [IQR 162–275 10^3^/mm^3^] and was higher in survivors compared with non-survivors only during the second and the third waves.

Plasm albumin levels were recorded in 3325 patients, with a median value of 32 g/L [IQR 28–35 g/L]. A lower level of plasm albumin was correlated with poorer survival.

**Fig. 2:**
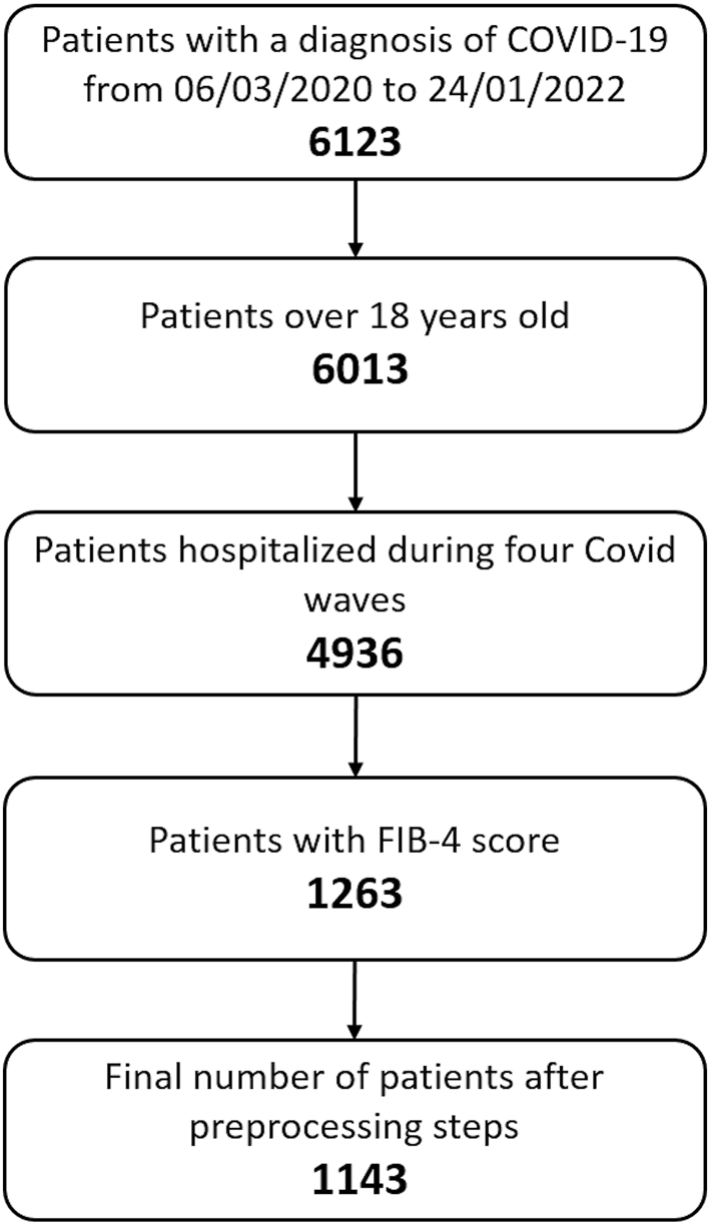
Flowchart of the study

The median of the FIB-4 score was 1.94 [IQR 1.16–3.36]. FIB-4 was available only for 1263/4936 (25.6%) patients (Fig. 2).

Supplementary Table 1 shows comparisons between patients for whom FIB-4 could be calculated and patients without a FIB-4 measurement. The median age of patients with FIB-4 was 65 years [IQR 51–77], while the median age of patients without a FIB-4 measurement was 67 years [IQR 54–79 years], *p* < 0.01. Patients with FIB-4 had a higher incidence of gastrointestinal disease (*p* = 0.02), chronic liver disease (*p* = 0.04), cirrhosis (*p* < 0.01) and immunodeficiency (*p* 0.01). In terms of symptoms at admission, FIB-4 patients more often had fever, anosmia/dysgeusia, cough (*p* < 0.01) and dyspnea (*p* = 0.05). Platelets count and triglycerides were higher in patients without FIB-4 (*p* < 0.01 and *p* = 0.01) while alkaline phosphatase was lower (*p* < 0.01).

**Fig. 3:**
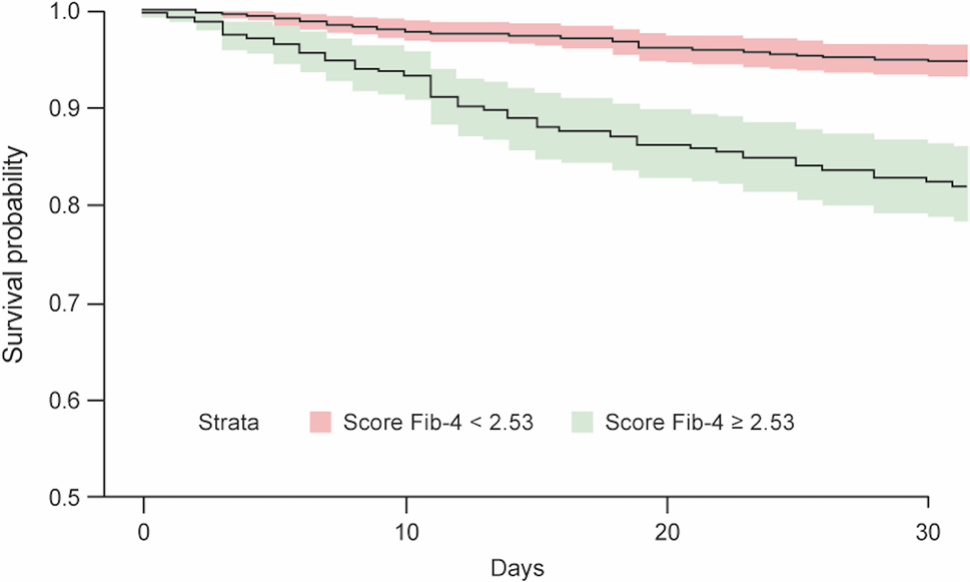
Kaplan–Meier survival probability curves with FIB-4 as covariate

Patients who died from COVID-19 had higher FIB-4 compared with those who were alive upon discharge in every single wave. FIB-4 score < 2.53 leads to a significant increment (*p* < 0.0001) in survival probability compared to a FIB-4 score ≥ 2.53 (Fig. 3).

**Fig. 4:**
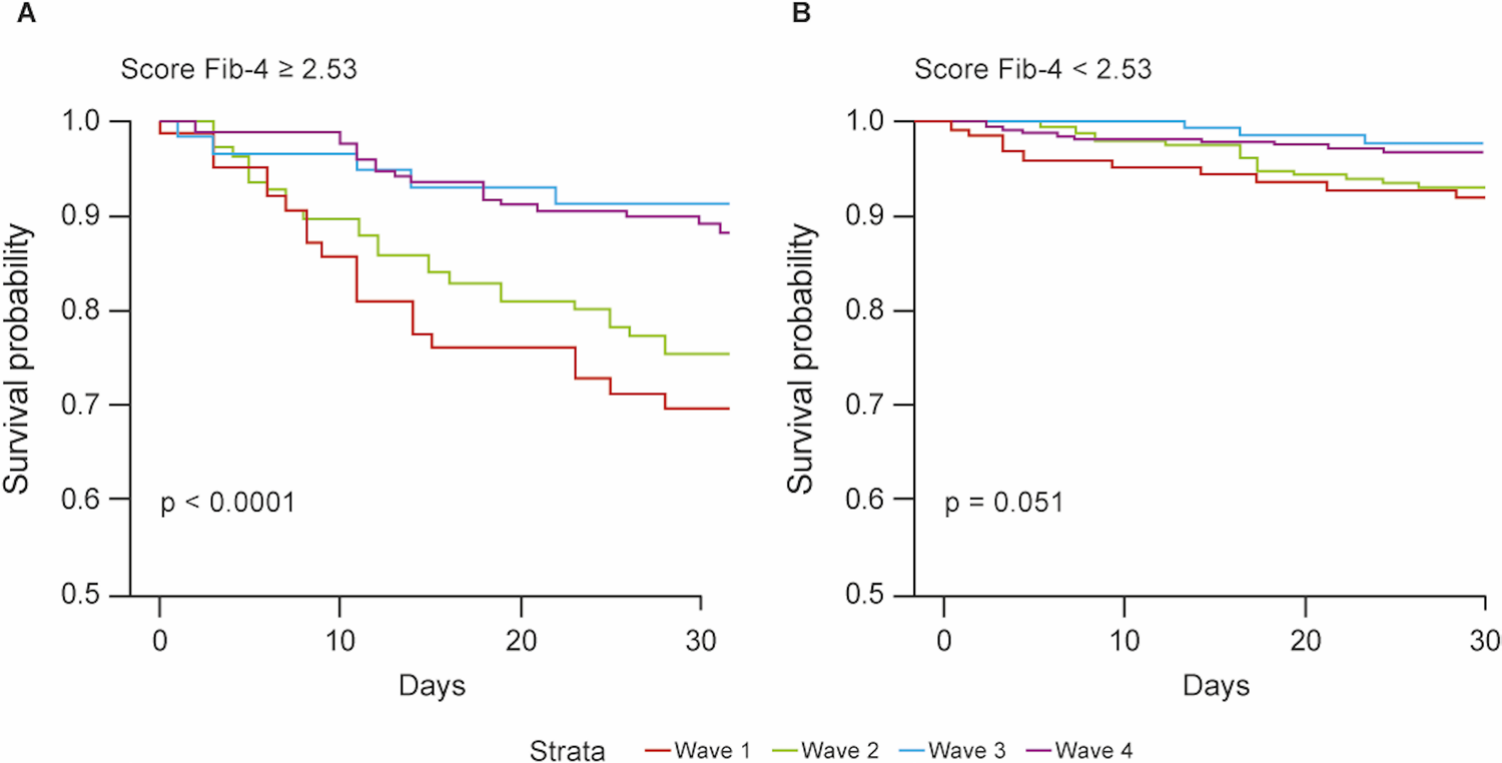
Kaplan–Meier survival probability curves across COVID waves for patients **A** with Fib-4 score < 2.53, **B** with Fib-4 score ≥ 2.53. *p*-values of the Log-rank test

Differences in COVID-19 waves mainly affect patients with high FIB-4 scores (≥ 2.53) (Fig. 4). Considering the group of patients with a high FIB-4 score, survival curves separated significantly across waves (*p* < 0.0001). A clear separation exists between the first/second waves, in which the probability of survival decreases rapidly over time, and the third/fourth waves, in which the probability of survival remains more constant over time.

In contrast, the separation between waves is not significant among patients with low FIB-4 scores (*p* = 0.051).

### Validation of a multivariable model for the primary outcome

**Fig. 5:**
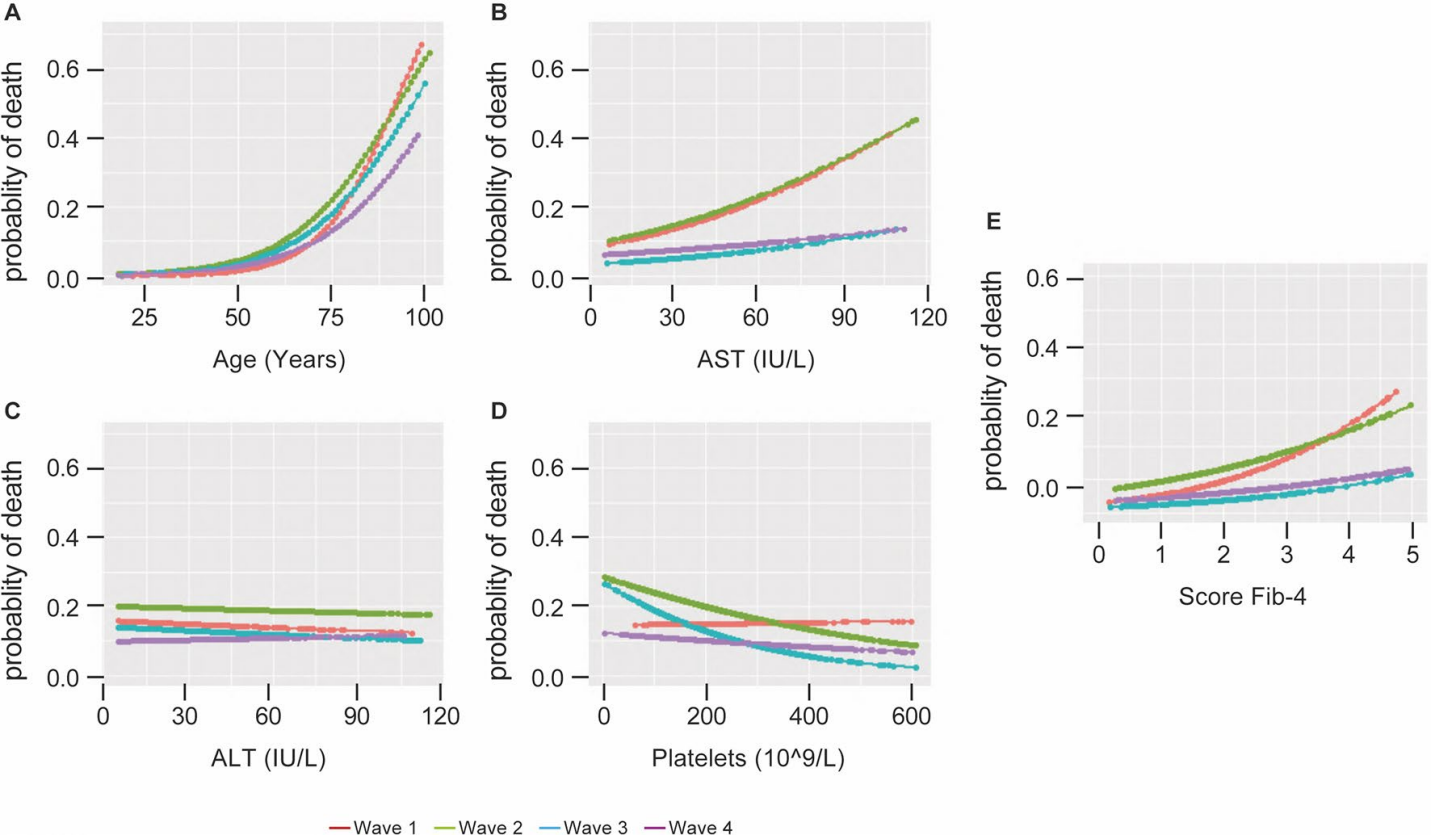
Univariate logistic regression curves. **A**–**D** show the probability of death versus Fib4 components. **E** shows the probability of death versus FIB-4. As shown in **B** and **E**, mortality risk curves are steeper during the first and second waves, the same AST and FIB-4 values are associated with a higher probability of death than in the third and fourth waves. The same behaviour does not emerge with the other FIB-4 components

The univariate logistic regression models fitted on the components of the FIB-4 score and the score itself are shown in Fig. 5. The relationship between the FIB-4 score and mortality risk is monotonically increasing with a steeper curve in the first and second waves.After preprocessing steps, 1143 patients and 35 variables were included in the final dataset. The whole sample was randomized in training (75% of the total number of observations) and in a testing sample (25% of the total number of observations) through a stratified sampling to maintain patient distributions across waves (16% during the first wave, 29% during the second wave, 15% during the third wave, 40% during the fourth wave).

The number of patients and the percentage of events in the training and test set were 856 (11.4%) and 287 (10.4%), respectively.

The binomial logistic regression shows that the mortality risk increases for FIB-4 score values ≥ 2.53 (OR = 4.53, 95% CI 2.83–7.25; *p* ≤ 0.001). Patients during the third wave (OR = 0.34, 95% CI 0.15–0.75; *p* = 0.007) and the fourth wave (OR = 0.40, 95% CI 0.24–0.66; *p* ≤ 0.001) had a decreased risk of mortality compared with other patients. The model also showed that mortality risk increases as LDH increases (OR = 1.001, 95% CI 1.000–1.002; *p* = 0.021). ROC curve analysis showed an AUC of 0.752 on the training set and 0.753 on the test set (Supplementary Fig. 1). The confusion matrix shows an accuracy of 0.76 (95% CI 0.70–0.81) with a sensitivity and specificity of 0.64 and 0.77 on the test set, respectively. Negative and positive predictive values are 0.94 and 0.25, respectively.

### Secondary outcomes (mechanical ventilation, ICU admission, LOS)

Patients requiring mechanical ventilation were older compared with those who did not (72 years [IQR 63–78 years] vs 68 years [IQR 53–80 years], *p* < 0.05) (Supplementary Tables 3 and 4).

Lower oxygen saturation was also associated with the need for mechanical ventilation (Supplementary Table 3), ICU admission (Supplementary Table 4), mortality (Table 1) and a lower probability of being discharged within 10 days (Supplementary Table 5). Furthermore, lower PLT count was correlated with admission to the ICU only during the second wave. There were no significant differences between the group who required mechanical ventilation and those who did not for the first and fourth waves. Patients admitted to ICU had similar FIB-4 scores compared with those who were not admitted to ICU (median 2.03 [IQR 1.45–3.90] vs 1.76 [IQR 0.98–3.19], *p* = 0.21). Patients who required mechanical ventilation had higher FIB-4 scores as compared to those who did not (3.15 [IQR 1.56–5.87] vs 1.81 [IQR 1.01–3.26]; *p* = 0.02). Lower FIB-4 were more likely to be discharged within 10 days when compared with patients with high FIB-4 (median 1.30 [IQR 0.74–2.36] vs 2.15 [IQR 1.41–4.00], *p* ≤ 0.01).

## Discussion

Our study shows that FIB-4, a simple score based on clinical data derived from routine laboratory analyses upon admission, is correlated with mortality and morbidity in patients with COVID-19. Although this parameter was available only for a proportion of patients, we also show the association of FIB-4 with COVID outcomes across four waves of infection.

Prognostic models for patients with COVID-19 [19–21], include factors, such as vital signs, age, comorbidities, and radiological features. Our study tested NLR, LDH, BUN, sodium, calcium, age, hemoglobin, and FIB-4 as independent risk factors for poorer outcomes.

FIB-4 has been validated for predicting the risk of fibrosis in liver disease and is recommended as a first-line, non-invasive test to rule out fibrosis [18]. Liver function tests alteration is frequently reported during SARS-CoV-2 infection and is probably due to direct viral damage to hepatocytes, cytokine release, ischemic liver damage or drug-induced liver injury [22, 23].

In univariate analysis, age was associated with a greater need for mechanical ventilation, LOS and reduced survival. Low PLT was also associated with reduced survival. Regarding transaminases, we found that higher values were related to a greater risk of mechanical ventilation for both ALT and AST. While ALT was related to ICU admission, AST was associated with prolonged LOS. Although we found no difference in mortality associated with transaminases, we disclosed a difference in FIB-4, resulting from the combination of age, ALT, AST and PLT count.

Our results are consistent with other studies that assessed the association of FIB-4 with mortality [2, 24], ICU admission and mechanical ventilation in COVID-19 patients [6, 10, 11].

The meaning of the FIB-4 score in COVID cohorts is still under debate. FIB-4 now appears to have relevance as a biomarker beyond the correlation with liver fibrosis or damage, especially in the case of SARS-CoV-2 infection. Some authors speculated that an elevated FIB-4 could reveal not only underlying liver disease but can reflect a “systemic” or multiorgan involvement of COVID-19 [7]. We agree with this speculation, but further studies are needed to understand the possible mechanisms supporting this hypothesis.

Our AI-based approach allowed us to test the variability over the four COVID-19 waves. Interestingly, among the four variables of FIB-4 score, age and AST (particularly in the first two waves) showed the most prognostic impact on mortality from COVID-19 infection, while ALT and PLT count have a minor but still significant role. Despite the prognostic role of age is well-established, AST role needs further discussion. In SARS-COV-2-related disease, AST elevation could either reflect direct hepatocellular damage or systemic inflammatory syndrome involving the liver and muscles. The hepatotropism of SARS-CoV-2 is further exacerbated in chronic liver diseases due to a higher expression of ACE2 receptors as a response to liver fibrosis [25, 26]. An elevation of AST in subjects with SARS-CoV-2 infection without chronic liver diseases could be related to direct cytotoxic damage and systemic and local pro-inflammatory responses. Interestingly, AST and ALT elevations are likely to persist after infection recovery, indicating chronic liver damage that could eventually lead to fibrosis and chronic liver disease [22, 27]. Alterations in liver enzymes are frequent in patients hospitalized for COVID-19, but the trajectory of alterations recorded during hospitalization is not always defined [28], and there is often a lack of data about pre-existing liver disease. The possible influence of such alterations on COVID-related mortality is a matter of debate. The EASL (European Association for the Study of the Liver) recently recommended the need for liver enzyme monitoring in patients hospitalized for hospitalized patients SARS-COV2 infection [29]. Further studies are needed to better understand the short- and long-term prognostic role of AST and ALT elevation in COVID-19-related disease.

Using a machine learning approach, we outlined a cut-off of 2.53 for FIB-4, beyond which the risk of death increases significantly. There have also been other studies that have considered FIB-4 in their models but with different cut-offs. Park et al. [7] found a cut-off of 4.95 to be a good predictor of mortality. Lombardi et al. [30] recently confirmed the prognostic role of FIB-4 in 382 patients. They showed that a FIB4 < 1.45 is a protective factor against severe SARS-CoV-2 infection and that in patients with at least one metabolic comorbidity, FIB-4 > 1.45 is associated with poorer outcomes. Bucci et al. [31] showed in a prospective cohort of patients that a FIB-4 cut-off of 2.76 has the best prognostic performance for survival in severe COVID-19. The strength of our cut-off is that it has been internally validated by examining the model’s performance in the training and test set, while the confusion matrix showed a well balanced accuracy.

This study has some limitations. First of all, it is a retrospective study design. Second, the AST assessment is not done routinely in the Emergency Department of our hospital, thus implying the reduction of the availability of data on FIB-4. The hospital’s policy of not providing routine AST determination is based on several factors. These include the limited diagnostic value of AST compared to other liver function tests such as ALT and ALP, which are more specific for liver damage in the emergency room setting. Additionally, the high variability of AST and its relatively higher cost compared to other liver function tests have also been considered. The decision is also in line with the recommendations from scientific societies that prioritize ALT over AST measurement in emergency room settings to assess liver function, as ALT is more specific to liver injury [32, 33]. Third, we were unaware of any previous pharmacological treatments that could potentially contribute to the elevation of liver enzymes before hospital admission. However, this limitation is, in our opinion, overcome by a large number of cases from a single center, which reduces the potential variability associated with drug use prior to hospitalization. Additionally, we did not have access to laboratory and anamnestic data related to underlying liver disease prior to SARS-CoV-2 infection. The strength of our study is the use of AI in collecting demographic and clinical data from each patient’s clinical diary. Another important point to note is that our study is monocentric, which eliminates the bias between different laboratory samplings.

The identification of a rapid, non-invasive, and cost-effective predictor of severe disease that could help in the early identification of patients who require more intensive monitoring would be of major clinical value. Indeed, this tool might facilitate the identification of cases at a higher risk of COVID-19-related clinical outcomes. Given the integration and automated data processing feature, the AI integration with electronic health records can be used for decision-support with a machine-based triage process that can help wards and Emergency Departments during periods of high peak and workloads. In addition, the availability of a patient-centered data set that is constantly updated with new patients and new clinical and laboratory data allows continuous learning and validation, with the potential to identify structural modification in the disease patterns, including the influence of variants and vaccines.

## Conclusion

In a large monocentric cohort of COVID-19 patients, we showed that FIB-4 assessed at hospital admission could provide prognostic information and help clinicians identify patients with COVID-19 disease at risk of in-hospital mortality, mechanical ventilation, and ICU admission.

FIB-4 could be an easy and inexpensive tool for stratification of the risk for COVID-19 subjects. The evaluation of FIB-4 routinely at patient admission could facilitate risk stratification and optimization of healthcare resources.

Given the specific setup, with predictors being developed and validated on a continuously updated patients cohort, we also show a paradigm for the integration in clinical practice of pragmatic, replicable decision support, enabling rapid assessment of disease severity at baseline and data-driven comparison over time of evolving disease patterns.

## Supplementary Information

Below is the link to the electronic supplementary material.

Supplementary file1 (DOCX 89 KB)

Supplementary file2 (DOCX 19 KB)

Supplementary file3 (DOCX 15 KB)

Supplementary file4 (TIF 756 KB)

Supplementary file5 (RTF 1167 KB)

## Data Availability

Data are available on request due to privacy/ethical restrictions.

## Abbreviations

FIB-4: Fibrosis-4 Index
GGRWD: Gemelli Generator Real World Data
ICU: Intensive care unit
LOS: Length of hospital stay
PLT: Platelets
ROC: Receiver operating characteristic
SME: Subject matter experts
