# Supplementary material for: Fib-4 score is able to predict intra-hospital mortality in 4 different SARS-COV2 waves
Source: Intern Emerg Med. 2023 Jul 25;18(5):1415–27. doi: 10.1007/s11739-023-03310-y (PMC10412472; doi:10.1007/s11739-023-03310-y)
Supplement: Supplementary file 2 — Supplementary file2 (DOCX 19 KB) [file 11739_2023_3310_MOESM2_ESM.docx]

**Appendix**

**Gemelli against COVID group:**

Abbate Valeria

Acampora Nicola

Addolorato Giovanni

Agostini Fabiana

Ainora Maria Elena

Airola Carlo

Akacha Karim

Amato Elena

Andreani Francesca

Andriollo Gloria

Annetta Maria Giuseppina

Annicchiarico Brigida Eleonora

Antonelli Mariangela

Antonelli Massimo

Antonucci Gabriele

Anzellotti Gian Marco

Armuzzi Alessandro

Baldi Fabiana

Barattucci Ilaria

Barillaro Christian

Barone Fabiana

Bellantone Rocco Domenico Alfonso

Bellieni Andrea

Bello Giuseppe

Benicchi Andrea

Benvenuto Francesca

Berardini Ludovica

Berloco Filippo

Bernabei Roberto

Bianchi Antonio

Biasucci Daniele Guerino

Biasucci Luigi Marzio

Bibbò Stefano

Bini Alessandra

Biolato Marco

Bisanti Alessandra

Biscetti Federico

Bocci Maria Grazia

Bonadia Nicola

Bongiovanni Filippo

Borghetti Alberto

Borriello Raffaele

Bosco Giulia

Bosello Silvia

Bove Vincenzo

Bramato Giulia

Brandi Vincenzo

Bruni Teresa

Bruno Carmine

Bruno Dario

Bungaro Maria Chiara

Buonomo Alessandro

Burzo Livia

Calabrese Angelo

Calvello Maria Rosaria

Calvez Valentino

Cambieri Andrea

Cambise Chiara

Cammà Giulia

Candelli Marcello

Canistro Gennaro

Cantanale Antonello

Capalbo Gennaro

Capaldi Lorenzo

Capannoli Luigi

Capone Emanuele

Capristo Esmeralda

Carbone Luigi

Cardone Silvia

Carelli Simone

Carfì Angelo

Carnicelli Annamaria

Caruso Cristiano

Casciaro Francesco Antonio

Catalano Lucio

Cauda Roberto

Cavallaro Chiara

Cecchini Andrea Leonardo

Cerniglia Giuseppe

Cerrito Lucia

Cesarano Melania

Chiarito Annalisa

Cianci Rossella

Cicchinelli Sara

Ciccullo Arturo

Cicetti Marta

Ciciarello Francesca

Cingolani Antonella

Cipriani Maria Camilla

Consalvo Maria Ludovica

Coppola Gaetano

Corbo Giuseppe Maria

Corsello Andrea

Costante Federico

Costanzi Matteo

Covino Marcello

Crupi Davide

Cutuli Salvatore Lucio

D'Addio Stefano

D'Alessandro Alessia

D'Alfonso Maria Elena

D’Amore Alessandra

D'Angelo Emanuela

D'Aversa Francesca

Damiano Fernando

De Berardinis Gian Maria

De Cunzo Tommaso

De Gaetano Donati Katleen

De Luca Giulio

De Matteis Giuseppe

De Pascale Gennaro

De Santis Paolo

De Siena Martina

De Vito Francesco

Del Gatto Valeria

Del Giacomo Paola

Del Zompo Fabio

Dell'Anna Antonio Maria

Della Polla Davide

Di Gialleonardo Luca

Di Giambenedetto Simona

Di Luca Roberta

Di Maurizio Luca

Di Muro Mariangela

Dusina Alex

Eleuteri Davide

Esperide Alessandra

Fachechi Daniele

Faliero Domenico

Falsiroli Cinzia

Fantoni Massimo

Fedele Annalaura

Feliciani Daniela

Ferrante Cristina

Ferrone Giuliano

Festa Rossano

Fiore Maria Chiara

Flex Andrea

Forte Evelina

Franceschi Francesco

Francesconi Alessandra

Franza Laura

Funaro Barbara

Fuorlo Mariella

Fusco Domenico

Gabrielli Maurizio

Gaetani Eleonora

Galletta Claudia

Gallo Antonella

Gambassi Giovanni

Garcovich Matteo

Gasbarrini Antonio

Gasparrini Irene

Gelli Silvia

Giampietro Antonella

Gigante Laura

Giuliano Gabriele

Giuliano Giorgia

Giupponi Bianca

Gremese Elisa

Grieco Domenico Luca

Guerrera Manuel

Guglielmi Valeria

Guidone Caterina

Gullì Antonio

Iaconelli Amerigo

Iafrati Aurora

Ianiro Gianluca

Iaquinta Angela

Impagnatiello Michele

Inchingolo Riccardo

Intini Enrica

Iorio Raffaele

Izzi Immacolata Maria

Jovanovic Tamara

Kadhim Cristina

La Macchia Rosa

La Milia Daniele Ignazio

Landi Francesco

Landi Giovanni

Landi Rosario

Landolfi Raffaele

Leo Massimo

Leone Paolo Maria

Levantesi Laura

Liguori Antonio

Liperoti Rosa

Lizzio Marco Maria

Lo Monaco Maria Rita

Locantore Pietro

Lombardi Francesco

Lombardi Gianmarco

Lopetuso Loris

Loria Valentina

Losito Angela Raffaella

Lucia Mothanje Barbara Patricia

Macagno Francesco

Macerola Noemi

Maggi Giampaolo

Maiuro Giuseppe

Mancarella Francesco

Mangiola Francesca

Manno Alberto

Marchesini Debora

Maresca Gian Marco

Marrone Giuseppe

Martis Ilaria

Martone Anna Maria

Marzetti Emanuele

Mattana Chiara

Matteo Maria Valeria

Maviglia Riccardo

Mazzarella Ada

Memoli Carmen

Miele Luca

Migneco Alessio

Mignini Irene

Milani Alessandro

Milardi Domenico

Montalto Massimo

Montemurro Giuliano

Monti Flavia

Montini Luca

Morena Tony Christian

Morra Vincenzina

Morretta Chiara

Moschese Davide

Murace Celeste Ambra

Murdolo Martina

Murri Rita

Napoli Marco

Nardella Elisabetta

Natalello Gerlando

Natalini Daniele

Navarra Simone Maria

Negri Marcantonio

Nesci Antonio

Nicoletti Alberto

Nicoletti Rocco

Nicoletti Tommaso Filippo

Nicolò Rebecca

Nicolotti Nicola

Nista Enrico Celestino

Nuzzo Eugenia

Oggiano Marco

Ojetti Veronica

Pagano Francesco Cosimo

Paiano Gianfranco

Pais Cristina

Pallavicini Federico

Palombo Andrea

Paolillo Federico

Papa Alfredo

Papanice Domenico

Papparella Luigi Giovanni

Paratore Mattia

Parrinello Giuseppe

Pasciuto Giuliana

Pasculli Pierpaolo

Pecorini Giovanni

Perniola Simone

Pero Erika

Petricca Luca

Petrucci Martina

Picarelli Chiara

Piccioni Andrea

Piccolo Annalisa

Piervincenzi Edoardo

Pignataro Giulia

Pignataro Raffaele

Pintaudi Gabriele

Pisapia Luca

Pizzoferrato Marco

Pizzolante Fabrizio

Pola Roberto

Policola Caterina

Pompili Maurizio

Pontecorvi Flavia

Pontecorvi Valerio

Ponziani Francesca

Popolla Valentina

Porceddu Enrica

Porfidia Angelo

Porro Lucia Maria

Potenza Annalisa

Pozzana Francesca

Privitera Giuseppe

Pugliese Daniela

Pulcini Gabriele

Racco Simona

Raffaelli Francesca

Ramunno Vittoria

Rapaccini Gian Ludovico

Richeldi Luca

Rinninella Emanuele

Rocchi Sara

Romanò Bruno

Romano Stefano

Rosa Federico

Rossi Laura

Rossi Raimondo

Rossini Enrica

Rota Elisabetta

Rovedi Fabiana

Rubino Carlotta

Rumi Gabriele

Russo Andrea

Russo Andrea

Sabia Luca

Salerno Andrea

Salini Sara

Salvatore Lucia

Samori Dehara

Sandroni Claudio

Sanguinetti Maurizio

Santarelli Luca

Santini Paolo

Santolamazza Danilo

Santoliquido Angelo

Santopaolo Francesco

Santoro Luca

Santoro Michele Cosimo

Sardeo Francesco

Sarnari Caterina

Saviano Angela

Saviano Luisa

Scaldaferri Franco

Scarascia Roberta

Schepis Tommaso

Schiavello Francesca

Scoppettuolo Giancarlo

Sedda Davide

Sessa Flaminio

Sestito Luisa

Settanni Carlo

Siciliano Matteo

Siciliano Valentina

Sicuranza Rossella

Simeoni Benedetta

Simonetti Jacopo

Smargiassi Andrea

Soave Paolo Maurizio

Sonnino Chiara

Spinelli Irene

Staiti Domenico

Stella Claudia

Stella Leonardo

Stival Eleonora

Taddei Eleonora

Talerico Rossella

Tamburello Elio

Tamburrini Enrica

Tanzarella Eloisa Sofia

Tarascio Elena

Tarli Claudia

Tersali Alessandra

Tilli Pietro

Timpano Jacopo

Torelli Enrico

Torrini Flavia

Tosato Matteo

Tosoni Alberto

Tricoli Luca

Tritto Marcello

Tumbarello Mario

Tummolo Anita Maria

Vallecoccia Maria Sole

Valletta Federico

Varone Francesco

Vassalli Francesco

Ventura Giulio

Verardi Lucrezia

Vetrone Lorenzo Maria

Vetrugno Giuseppe

Visconti Elena

Visconti Felicia

Viviani Andrea

Zaccaria Raffaella

Zaccone Carmelina

Zelano Lorenzo

Zileri Dal Verme Lorenzo

Zuccalà Giuseppe
