# Supplementary material for: Fib-4 score is able to predict intra-hospital mortality in 4 different SARS-COV2 waves
Source: Intern Emerg Med. 2023 Jul 25;18(5):1415–27. doi: 10.1007/s11739-023-03310-y (PMC10412472; doi:10.1007/s11739-023-03310-y)
Supplement: Supplementary file 3 — Supplementary file3 (DOCX 15 KB) [file 11739_2023_3310_MOESM3_ESM.docx]

**Supplementary material**

*Extraction of clinical data*

Clinical data were extracted from both structured and unstructured data sources. Text mining techniques were employed to obtain structured information from unstructured data sources automatically: comorbidities were extracted from clinical charts and discharge letters, while symptoms were extracted from emergency room records and clinical charts.

Included symptoms were fever, anosmia/dysgeusia, cough, dyspnea, myalgia/arthralgia, and gastrointestinal symptoms. Vital signs included: oxygen saturation, body temperature, cardiac frequency, respiratory rate per minute, mean blood pressure, and PaO_2_/FiO_2_ ratio. The following laboratory exams were collected: complete blood count, D-dimer, procalcitonin, interleukin, HS troponin, lactate, glucose, creatinine, ALT, AST, GGT, ALP, bilirubin, albumin, cholesterol (total and HDL), triglycerides and international normalized ratio (INR). The neutrophil-to-lymphocyte ratio (NLR) was calculated as the ratio between the neutrophil and lymphocyte counts in peripheral blood.

The collected comorbidities were obesity, diabetes, hypertension, malignancy, neurological disease, stroke, cardiovascular disease, pneumopathy, gastrointestinal disease, chronic kidney disease, chronic liver disease, cirrhosis, thrombosis, and immunodeficiency.

*Stratified sampling strategy*

A stratified sampling strategy was adopted to preserve patient distributions across waves.

Before model fitting, some pre-processing steps were performed. First, all variables with p<0.01 after the univariate analysis on in-hospital mortality were considered. Of these variables: i) all those with a percentage of missing values >30% were not considered; ii) all rows containing missing values were deleted from the final dataset; iii) to increase model performance, some of the continuous variables (NLR score, FIB-4 score, hemoglobin, hematocrit, calcium, urea nitrogen and Charlson score) were discretized through a cut-off detection algorithm.

The algorithm cycled through the available variables one by one and independently divided them into a varying number of equal-sized buckets to assess the presence/absence of a linear correlation with the selected outcomes. All variables were initialized with a default value of eight separate buckets, and these buckets were then successively used as dummy variables in univariate logistic regression. The significance of the separation between the different buckets was quantified through the coefficients p-values. Based on the resulting p-values and coefficients, the linearity and direction of the correlation of the single buckets with the outcome could be extrapolated. Additionally, if the results indicated a non-significant separation between the generated buckets, the number of buckets was reduced by one, and the algorithm was restarted for a new cycle to determine the maximum number of significant buckets still indicating separations between the single buckets and clear correlation indications, all still retaining statistical significance. The resulting cut-offs were then used in order to discretize the respective variables.

## **Supplementary Figure 1.** Receiver operating characteristic (ROC) curves on training and test set for logistic regression model on survival outcome
