# Supplementary figures and images for: Fib-4 score is able to predict intra-hospital mortality in 4 different SARS-COV2 waves
Source: Intern Emerg Med. 2023 Jul 25;18(5):1415–27. doi: 10.1007/s11739-023-03310-y (PMC10412472; doi:10.1007/s11739-023-03310-y)

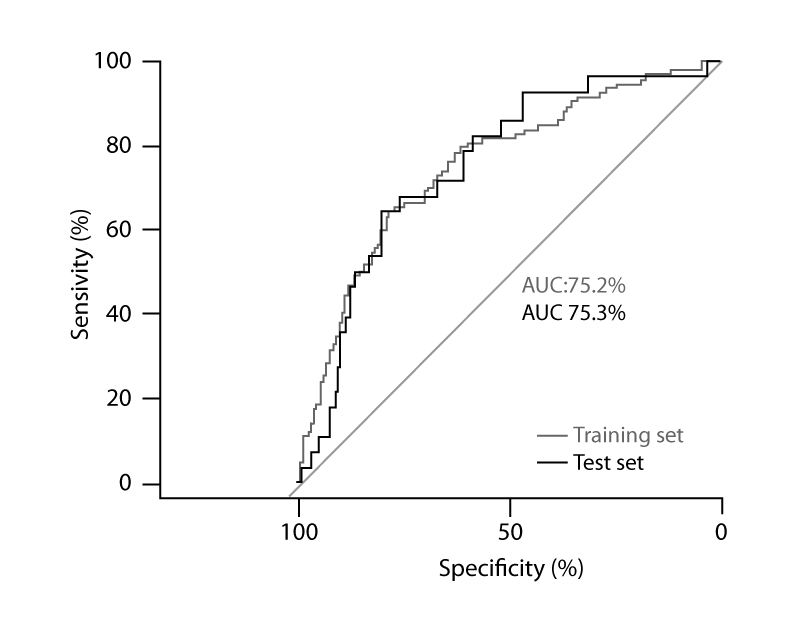

Supplement: Supplementary file 4 — Supplementary file4 (TIF 756 KB) [file 11739_2023_3310_MOESM4_ESM.tif]
