# Supplementary material for: Fib-4 score is able to predict intra-hospital mortality in 4 different SARS-COV2 waves
Source: Intern Emerg Med. 2023 Jul 25;18(5):1415–27. doi: 10.1007/s11739-023-03310-y (PMC10412472; doi:10.1007/s11739-023-03310-y)
Supplement: Supplementary file 5 — Supplementary file5 (RTF 1167 KB) [file 11739_2023_3310_MOESM5_ESM.rtf]

Supplementary Table 1. Demographic and clinical data in the general population and comparison of patients with and without Fib4 measurement.
	Total† population	Fib4† population	Non-Fib4† population	p-value	
Demographics	
No. patients	4936	1263	3673		
Wave:					
1	570 (11.55)	201 (15.91)	369 (10.05)		
2	2336 (47.33)	360 (28.50)	1976 (53.80)	<0.01	
3	1064 (21.56)	196 (15.52)	868 (23.63)		
4	966 (19.57)	506 (40.06)	460 (12.52)		
Age (years)	67 (53–78)	65 (51–77)	67 (54–79)	<0.01	
Male	2707 (54.84)	700 (55.42)	2007 (54.64)	0.65	
BMI (kg/m2)	26.12 (23.66–29.38)	26.12 (23.53–29.39)	26.12 (23.67–29.38)	0.87	
Comorbidities	
Obesity (BMI >30 kg/m2)	880 (22.37)	248 (23.40)	632 (21.99)	0.37	
Diabetes	800 (16.34)	192 (15.21)	608 (16.73)	0.23	
Hypertension	1878 (38.35)	455 (36.05)	1423 (39.15)	0.06	
Malignancy	595 (12.15)	153 (12.12)	442 (12.16)	1	
Neurological disease	267 (5.45)	79 (6.26)	188 (5.17)	0.16	
Stroke	209 (4.23)	56 (4.43)	153 (4.17)	0.74	
Cardiovascular disease	341 (6.96)	85 (6.74)	256 (7.04)	0.76	
Pneumopathy	485 (9.9)	137 (10.86)	348 (9.57)	0.21	
Gastrointestinal disease	108 (2.21)	39 (3.09)	69 (1.9)	0.02	
Chronic kidney disease	319 (6.51)	94 (7.45)	225 (6.19)	0.14	
Chronic liver disease	54 (1.1)	21 (1.66)	33 (0.91)	0.04	
Cirrhosis	55 (1.12)	33 (2.61)	22 (0.61)	<0.01	
Thrombosis/pulmonary embolism	117 (2.37)	31 (2.45)	86 (2.34)	0.9	
Immunodeficiency	126 (2.57)	45 (3.57)	81 (2.23)	0.01	
Symptoms at admission	
Fever	1871 (61.89)	543 (66.14)	1328 (60.31)	<0.01	
Anosmia/dysgeusia	109 (4.1)	49 (6.82)	60 (3.1)	<0.01	
Cough	931 (32.67)	293 (37.95)	638 (30.7)	<0.01	
Dyspnea	1576 (53.9)	461 (56.91)	1115 (52.74)	0.05	
Myalgia/arthralgia	503 (17.98)	146 (19.34)	357 (17.48)	0.28	
Gastrointestinal	228 (8.39)	67 (9.14)	161 (8.12)	0.44	
Hospital admission data	
Oxygen saturation (%)	96.00 (94.00–97.00)	96.00 (94.00–97.00)	96.00 (94.00–97.00)	<0.01	
Body temperature, °C	36.30 (36.00–37.50)	36.30 (36.00–37.50)	36.30 (36.00–37.50)	0.87	
Cardiac Frequency (bpm)	80.00 (72.00–89.00)	80.00 (72.00–89.00)	80.00 (72.00–89.00)	0.61	
Respiratory (rate per minute)	14 (12–15)	14 (13–15)	13 (12–15)	<0.01	
Mean blood pressure (mmHg)	90 (83–100)	92 (83–100)	90 (83–100)	0.18	
P/F	217.90 (147.33–319.00)	221.40 (149.30–321.00)	217.10 (146.30–318.80)	0.55	
Laboratory tests	
Hemoglobin (g/dL)	13.30 (11.70–14.60)	13.30 (11.70–14.50)	13.25 (11.70–14.60)	0.63	
Haematocrit (%)	39.00 (34.80–42.70)	39.00 (34.70–42.20)	39.00 (34.90–42.90)	0.3	
Platelets (cell × 109/L)	211.00 (162.00–275.00)	201.00 (153.00–265.00)	214.00 (165.00–279.00)	<0.01	
WBC (cell × 109/L)	7.62 (5.52–10.63)	7.34 (5.39–10.20)	7.76 (5.57–10.77)	0.01	
Neutrophils (cell × 109/L)	5.82 (3.96–8.58)	5.56 (3.82–8.07)	5.93 (4.01–8.81)	<0.01	
Lymphocytes (cell × 109/L)	1.03 (0.72–1.50)	1.04 (0.75–1.55)	1.03 (0.71–1.48)	0.12	
D-dimer (ng/mL)	896.00 (483.00–2003.00)	897.00 (502.00–2008.00)	895.00 (475.00–2002.00)	0.35	
Procalcitonin (ng/mL)	0.13 (0.07–0.31)	0.13 (0.08–0.34)	0.12 (0.07–0.30)	0.06	
IL-6 (ng/L)	20.85 (9.17–44.00)	22.00 (9.70–48.10)	20.30 (9.00–42.30)	0.21	
HS troponin (ng/L)	13.00 (6.00–35.00)	13.00 (6.00–34.00)	12.00 (6.00–36.00)	0.81	
Lactate (mmol/L)	1.00 (0.80–1.50)	1.00 (0.70–1.40)	1.10 (0.80–1.50)	<0.01	
Lactic dehydrogenase (IU/L)	310.00 (229.75–434.00)	308.00 (225.00–426.00)	311.00 (232.00–437.75)	0.15	
Glucose (mg/dL)	114.00 (97.00–142.00)	111.00 (95.00–135.00)	116.00 (98.00–145.00)	<0.01	
Creatinine (mg/dL)	0.87 (0.69–1.14)	0.87 (0.68–1.15)	0.87 (0.69–1.14)	0.67	
ALT (IU/L)	24.00 (15.00–40.00)	24.00 (15.00–43.00)	24.00 (15.00–39.00)	0.03	
AST (IU/L)	31.00 (22.00–49.00)	31.00 (22.00–49.00)	24.00 (19.50–39.00)	0.14	
GGT (IU/L)	34.00 (20.00–62.00)	34.00 (20.00–62.00)	34.00 (21.00–62.75)	0.81	
ALP (IU/L)	64.00 (50.00–87.00)	66.00 (51.25–92.75)	63.00 (50.00–84.25)	<0.01	
Total bilirubin (mg/dL)	0.60 (0.40–0.80)	0.60 (0.50–0.90)	0.60 (0.40–0.80)	0.02	
Direct bilirubin (mg/dL)	0.60 (0.40–1.10)	0.70 (0.50–1.50)	0.60 (0.40–0.80)	<0.01	
Albumin (g/L)	32.00 (28.00–35.00)	33.00 (29.00–37.00)	31.00 (28.00–35.00)	<0.01	
Total cholesterol (mg/dL)	139.00 (116.00–165.00)	136.00 (113.00–165.00)	140.00 (117.00–164.00)	0.28	
HDL cholesterol (mg/dL)	33.00 (26.00–42.00)	33.00 (27.00–42.00)	33.00 (26.00–41.00)	0.46	
Triglycerides (mg/dL)	118.00 (91.00–156.00)	112.00 (89.00–152.00)	121.00 (92.00–157.00)	0.01	
INR	1.04 (0.99–1.11)	1.03 (0.98–1.11)	1.04 (0.99–1.11)	<0.01	
Noninvasive tests	
FIB-4	1.94 (1.16–3.36)	1.94 (1.16–3.36)	–		
Clinical outcomes					
Length of stay (days)	11.00 (6.00–20.00)	11.00 (6.00–20.00)	11.00 (6.00–20.00)	0.48	
Discharge within 10 days	1981 (40.13)	526 (41.65)	1455 (39.66)	0.23	
ICU admission	1178 (23.87)	253 (20.03)	925 (25.18)	<0.01	
Mechanical ventilation	624 (12.64)	155 (12.27)	469 (12.77)	0.68	
In-hospital death	762 (15.44)	156 (12.35)	606 (16.5)	<0.01	
†Values shown are the median (IQR) for quantitative variables and n (%) for categorical variables.
‡Fib4 vs. non-Fib4 subgroups. Quantitative variables were compared through T-tests or Mann-Whitney U test. according to their distributions. Categorical variables were compared by Chi-squared test. Significance level ≤0.05
Supplementary Table 2. Patients' demographic characteristics and outcomes distribution in each pandemic wave
	Wave 1	Wave 2	Wave 3	Wave 4	p-value	
n	570	2336	1064	966		
Age (years)	69 (55–79)	69 (55–80)	64 (51–75)	64 (49–76)	<0.001	
Male	340 (59.65)	1269 (54.32)	600 (56.39)	498 (51.55)	0.01	
In-hospital mortality	85 (14.91)	448 (19.18)	133 (12.5)	96 (9.94)	<0.001	
Discharge in 10 days	150 (26.32)	916 (39.21)	449 (42.36)	466 (48.24)	<0.001	
Mechanical ventilation	82 (14.39)	281 (12.03)	158 (14.85)	103 (10.66)	0.01	
ICU	115 (20.18)	588 (25.17)	282 (26.5)	193 (19.98)	<0.001	
FIB-4	1.97 (1.21–3.30)	1.82 (1.02–3.26)	1.94 (1.06–3.18)	2.00 (1.23–3.57)	0.14	
Values shown are the median (IQR) for quantitative variables and number (percentage) for categorical variables.
Quantitative variables were compared through Kruskall-Wallis test. Categorical variables were compared by Chi-squared test. Significance level ≤0.05.
Supplementary Table 3. Mechanical ventilation along the four pandemic waves
	Wave 1 (n=570)	Wave 2 (n=2336)	Wave 3 (n=1064)	Wave 4 (n=966)	
	

No Mechanical ventilation	

Mechanical ventilation	p-value	

No Mechanical ventilation	

Mechanical ventilation	p-value	

No Mechanical ventilation	

Mechanical ventilation	p-value	

No Mechanical ventilation	

Mechanical ventilation	p-value	
Demographics	
No. patients	488	82		2055	281		906	158		863	103		
Age (years)	68 (54–80)	72 (63–7)	0.05	69 (54–81)	69 (60–75)	0.31	62 (49–75)	67 (57–74)	0.03	64 (47–77)	66 (57–74)	0.43	
Male	279 (57.1)	61 (74.3)	<0.01	1068 (51.9)	201 (71.5)	<0.01	487 (53.7)	113 (71.5)	<0.01	425 (49.2)	73 (70.8)	<0.01	
BMI (kg/m2)	25.25
(23.15–27.44)	27.08
(24.11–29.41)	0.01	25.88
(23.59–28.91)	27.04
(24.69–30.53)	<0.01	26.31
(23.88–29.76)	28.04
(24.91–32.65)	0.01	26.23
(23.38–29.99)	27.78
(25.25–31.35)	<0.01	
Comorbidities	
Obesity (BMI>30 kg/m2)	61 (13.38)	19 (23.17)	0.07	330 (20.55)	39 (26.53)	0.09	205 (24.67)	41 (36.28)	0.01	165 (25.08)	28 (36.36)	0.04	
Diabetes	67 (13.7)	11 (13.4)	1	364 (17.9)	63 (22.42)	0.08	113 (12.6)	27 (17)	0.13	132 (15.3)	23 (22.3)	0.09	
Hypertension	164 (33.6)	38 (46.3)	0.03	822 (40.5)	142 (50.5)	<0.01	320 (35.7)	58 (36.7)	0.86	292 (33.8)	42 (40.7)	0.19	
Malignancy	54 (11)	8 (9.7)	0.85	263 (12.9)	26 (9.2)	0.08	83 (9.2)	11 (6.9)	0.45	139 (16.1)	11 (10.6)	0.19	
Neurological disease	36 (7.3)	4 (4.8)	0.49	140 (6.9)	7 (2.4)	<0.01	33 (3.6)	2 (1.2)	0.15	44 (5.1)	1 (0.9)	0.08	
Stroke	17 (3.4)	4 (4.8)	0.53	107 (5.2)	23 (8.1)	0.05	28 (3)	5 (3.1)	1	20 (2.3)	5 (4.8)	0.18	
Cardiovascular disease	27 (5.5)	6 (7.3)	0.45	158 (7.7)	24 (8.5)	0.64	52 (5.8)	10 (6.3)	0.72	58 (6.7)	6 (5.8)	1	
Pneumopathy	64 (13.1)	8 (9.7)	0.48	196 (9.6)	45 (16)	<0.01	74 (8.2)	13 (8.2)	1	76 (8.8)	9 (8.7)	1	
Gastrointestinal disease	11 (2.2)	1 (1.2)	1	37 (1.8)	6 (2.1)	0.64	20 (2.2)	3 (1.9)	1	27 (3.)	3 (2.9)	1	
Chronic Kidney disease	24 (4.9)	2 (2.4)	0.56	136 (6.7)	25 (8.9)	0.17	35 (3.9)	6 (3.8)	1	81 (9.3)	10 (9.7)	0.86	
Chronic Liver disease	7 (1.4)	1 (1.2)	1	18 (0.8)	4 (1.4)	0.33	9 (1)	3 (1.9)	0.4	11 (1.2)	1 (0.9)	1	
Cirrhosis	1 (0.2)	0 (0)	1	21 (1)	4 (1.4)	0.54	7 (0.7)	0 (0)	0.6	17 (1.9)	5 (4.8)	0.08	
Thrombosis/pulmo- nary embolism	14 (2.8)	9 (10.9)	<0.01	44 (2.1)	4 (1.4)	0.65	26 (2.8)	5 (3.1)	0.8	13 (1.5)	2 (1.9)	0.67	
Immunodeficiency	10 (2)	1 (1.2)	1	38 (1.8)	14 (4.9)	<0.01	19 (2.1)	2 (1.2)	0.76	31 (3.5)	11 (10.6)	<0.01	
Symptoms at admission	
Fever	225 (77.8)	32 (71.1)	0.34	693 (59.2)	94 (58.)	0.86	459 (68)	59 (54.1)	0.01	280 (55.8)	29 (39.7)	0.01	
Anosmia/dysgeusia	14 (5.3)	1 (2.3)	0.7	62 (6.3)	1 (0.6)	<0.01	22 (3.5)	2 (1.7)	0.56	7 (1.6)	0 (0)	0.6	
Cough	145 (51.4)	18 (4)	0.2	326 (30.2)	46 (29.3)	0.85	211 (32.7)	20 (17)	<0.01	150 (32.7)	15 (20.5)	0.04	
Dyspnea	152 (53.3)	28 (59.5)	0.53	580 (51.8)	95 (62.9)	0.01	389 (59.3)	65 (59)	1	233 (47)	34 (48)	0.9	
Myalgia/arthralgia	46 (17.1)	12 (27.27)	0.14	189 (18.23)	19 (11.88)	0.06	140 (21.51)	12 (10.81)	0.01	80 (17.66)	5 (6.94)	0.02	
Gastrointestinal	38 (13.7)	5 (11.6)	0.81	89 (8.9)	9 (5.8)	0.28	52 (8.2)	5 (4.5)	0.24	29 (6.6)	1 (1.4)	0.1	
Hospital admission data	
Oxygen saturation (%)	96 (94–98)	94 (92–97)	<0.01	96 (94–97)	94 (92–97)	<0.01	96 (94–97)	95 (94–96)	<0.01	96 (95–98)	95.6 (94–96)	0.01	
Body temperature (°C)	37 (36–38)	38 (36.9–38.9)	<0.01	36.2 (36–37.2)	36.4 (36–37.7)	0.05	36.3 (36–37.5)	36.4 (36–37.7)	0.36	36.3 (36–37.3)	36.2 (36–37.7)	0.75	
Cardiac frequency (bpm)	79.5 (70.0–89.0)	83 (77–93)	0.02	80 (72–89)	79 (70–88)	0.58	80 (73–90)	80 (74–91)	0.63	80 (72–88)	80.5 (73.5–88.2)	0.69	
Respiratory (rate per minute)	13 (12–15)	14 (12–15)	0.66	13 (12–14)	13 (12–15)	0.47	14 (12–16)	14 (12.5–20)	0.02	14 (13–15)	15 (13–17)	0.01	
Mean blood pressure (mmHg)	90 (83–97)	93. (83–102)	0.07	90 (83–99)	90 (83–100)	0.64	93 (84–101)	93 (86–101)	0.3	91 (83–100)	92 (86–103)	0.63	
P/F	318.90
(212.98–378.08)	162.45
(117.53–223.32)	<0.01	229.65
(156.55–318.20)	127.30
(97.80–187.05)	<0.01	212.50
(151.50–299.45)	122.65
(99.70–171.05)	<0.01	208.60
(144.40–300.00)	130.00
(90.80–159.05)	<0.01	
Laboratory tests													
Hemoglobin (g/dl)	13.60
(12.20–14.80)	13.80
(12.00–14.80)	0.98	13.10
(11.50–14.40)	13.50
(11.80–14.80)	0.02	13.40
(12.10–14.80)	13.65
(11.80–14.80)	0.86	13.10
(11.50–14.50)	13.30
(11.50–15.00)	0.33	
Haematocrit (%)	40.30
(36.70–43.40)	40.20
(35.70–43.20)	0.64	38.80
(34.50–42.50)	39.90
(35.58–43.52)	0.01	39.20
(35.80–43.10)	39.90
(35.70–43.50)	0.55	37.90
(33.70–41.20)	37.80
(32.75–42.53)	0.79	
Platelets (cell × 109/l)	209.00
(165.00–270.00)	191.00
(151.00–262.00)	0.14	220.00
(171.00–285.00)	208.00
(154.00–275.50)	0.01	201.50
(159.00–266.75)	188.00
(143.25–243.75)	0.01	206.00
(155.00–267.50)	210.00
(149.25–289.75)	0.76	
WBC (cell × 109/l)	6.21 (4.84–8.65)	7.54 (5.38–9.62)	0.01	8.06 (5.83–11.07)	8.49 (5.94–11.36)	0.3	7.36 (5.27–10.06)	7.44 (5.33–10.61)	0.39	7.41 (5.52–10.62)	8.98 (5.78–13.73)	0.01	
Neutrophils (cell × 109/l)	4.63 (3.27–6.42)	6.29 (4.15–8.15)	<0.01	6.22 (4.20–9.09)	6.94 (4.57–9.77)	0.01	5.64 (3.82–8.09)	5.96 (4.11–9.21)	0.1	5.55 (3.96–8.27)	7.48 (4.61–12.40)	<0.01	
Lymphocytes (cell × 109/l)	1.13 (0.82–1.51)	0.86 (0.66–1.20)	<0.01	1.11 (0.78–1.57)	0.83 (0.57–1.27)	<0.01	0.99 (0.70–1.41)	0.78 (0.57–1.09)	<0.01	1.04 (0.73–1.56)	0.82 (0.64–1.18)	<0.01	
D-dimer (ng/ml)	934.00
(486.75–1881.75)	1470.50
(830.25–2999.00)	<0.01	899.00
(468.00–1997.00)	1041.00
(584.00–2468.00)	0.01	705.50
(414.75–1476.75)	984.00
(617.75–2596.25)	<0.01	938.00
(519.50–2209.00)	1380.00
(701.50–4198.50)	<0.01	
Procalcitonin (ng/mL)	0.12 (0.07–0.27)	0.14 (0.08–0.28)	0.21	0.12 (0.07–0.30)	0.19 (0.10–0.40)	<0.01	0.10 (0.07–0.19)	0.15 (0.08–0.35)	<0.01	0.14 (0.08–0.39)	0.17 (0.09–0.60)	0.25	
IL-6 (ng/l)	14.50
(7.73–29.58)	44.50
(21.50–73.90)	<0.01	19.60
(8.85–43.10)	25.90
(10.68–56.12)	0.03	19.40
(8.60–37.10)	33.10
(12.30–64.80)	<0.01	23.25
(10.35–51.15)	38.80
(17.50–84.70)	0.01	
HS troponin (ng/L)	10.00
(5.00–34.00)	23.00
(17.00–66.00)	<0.01	12.00
(6.00–34.00)	27.50
(9.00–93.75)	<0.01	9.00
(5.00–23.00)	19.00
(9.00–58.00)	<0.01	13.00
5.00–36.00)	20.00
(8.00–80.00)	0.04	
Lactate	1.15 (0.80–1.67)	1.00 (0.75–1.85)	0.8	1.10 (0.80–1.60)	1.20 (0.90–1.50)	0.15	1.00 (0.70–1.40)	1.10 (0.90–1.80)	0.02	1.00 (0.70–1.30)	1.00 (0.90–1.45)	0.19	
Lactic dehydrogenase (IU/l)	279.50
(216.00–393.00)	405.00
(321.50–645.00)	<0.01	295.00
(223.00–407.25)	439.00
(319.00–577.50)	<0.01	318.00
(236.00–428.00)	416.00
(300.50–602.50)	<0.01	294.00
(215.00–413.00)	402.00
(296.00–556.75)	<0.01	
Glucose (mg/dl)	107.00
(95.00–126.00)	119.00
(107.00–146.75)	<0.01	114.00
(96.00–142.00)	132.00
(111.00–169.00)	<0.01	113.00
(96.00–136.00)	131.00
(112.00–173.00)	<0.01	110.00
(96.00–135.00)	143.00
(121.00–222.00)	<0.01	
Creatinine (mg/dL)	0.89 (0.72–1.12)	0.95 (0.78–1.31)	0.02	0.87 (0.69–1.15)	0.99 (0.76–1.37)	<0.01	0.82 (0.66–1.01)	0.90 (0.73–1.14)	<0.01	0.88 (0.70–1.17)	1.04 (0.75–1.41)	0.03	
ALT (IU/l)	23.00
(15.00–38.00)	35.00
(18.00–50.00)	<0.01	22.00
(14.00–36.00)	31.00
(19.00–53.00)	<0.01	27.00
17.00–43.00)	31.00
(21.00–45.00)	0.01	21.00
(13.00–38.00)	29.00
(19.25–44.00)	<0.01	
AST (IU/l)	31.00
(23.00–49.25)	46.00
(32.50–63.50)	0.01	29.00
(20.00–45.00)	39.50
(26.25–74.75)	<0.01	31.00
(21.00–49.00)	44.00
(31.00–55.50)	0.04	30.00
(21.00–46.00)	44.50
(29.00–63.25)	<0.01	
GGT (IU/l)	31.00
(18.00–59.25)	47.50
(23.25–61.00)	0.09	31.00
(19.00–59.25)	46.00
(25.00–77.00)	<0.01	34.00
(21.00–61.00)	40.00
(25.00–73.00)	0.07	31.00
19.00–62.00)	33.00
(22.00–58.00)	0.42	
ALP (IU/l)	70.00
(55.00–89.00)	59.00
(48.00–79.00)	0.02	67.00
53.00–88.00)	60.00
(47.00–84.00)	0.01	60.00
48.00–80.25)	54.00
(44.00–73.75)	0.01	66.00
(52.75–98.00)	59.50
(49.00–91.50)	0.07	
Total bilirubin (mg/dL)	0.60 (0.40–0.80)	0.60 (0.40–0.90)	0.56	0.60 (0.40–0.80)	0.60 (0.50–0.90)	0.08	0.60 (0.50–0.80)	0.60 (0.40–0.80)	0.92	0.60 (0.40–0.80)	0.70 (0.50–1.00)	0.04	
Direct bilirubin (mg/dL)	0.50 (0.35–0.60)	0.45 (0.30–1.05)	0.77	0.60 (0.50–0.90)	0.80 (0.50–1.55)	0.06	0.60 (0.40–0.80)	0.60 (0.50–1.15)	0.25	0.60 (0.40–1.70)	1.20 (0.80–3.40)	0.08	
Albumin (g/l)	35.00
(31.00–38.00)	29.00
(27.00–32.50)	<0.01	32.00
(28.00–35.00)	29.00
(27.00–32.00)	<0.01	33.00
(30.00–35.00)	30.00
(27.00–33.00)	<0.01	32.00
(28.00–36.00)	29.00
(25.00–32.00)	<0.01	
Total cholesterol (mg/dl)	136.00
(115.00–170.25)	117.00
(92.50–146.00)	<0.01	144.00
(120.00–168.25)	126.00
(107.00–152.75)	<0.01	139.00
(118.00–166.00)	128.00
(107.25–152.00)	0.01	137.50
(113.00–165.00)	130.00
(107.00–149.00)	0.01	
HDL cholesterol (mg/dl)	31.00
(26.00–41.00)	22.00
(20.00–30.50)	<0.01	35.00
(27.00–42.00)	28.00
(20.00–35.00)	<0.01	33.00
(27.00–40.00)	30.00
(26.00–36.25)	0.08	35.00
(28.00–43.00)	27.00
(21.00–35.25)	<0.01	
Triglycerides (mg/dl)	108.50
(87.75–150.00)	152.00
(99.50–192.50)	0.04	118.00
(92.00–155.00)	134.50
(103.25–190.75)	<0.01	116.00
(91.00–149.00)	139.00
(105.50–176.50)	<0.01	109.00
(85.00–149.00)	141.00
(105.50–202.50)	<0.01	
INR	1.04 (1.00–1.09)	1.07 (1.02–1.12)	0.03	1.03 (0.98–1.09)	1.04 (0.99–1.14)	<0.01	1.07 (1.02–1.13)	1.09 (1.04–1.14)	0.01	1.02 (0.97–1.08)	1.06 (1.00–1.17)	<0.01	
Noninvasive tests	
FIB-4	1.86 (1.19–3.20)	2.63 (1.94–4.61)	0.01	1.72 (0.97–3.00)	2.87 (1.50–4.09)	<0.01	1.86 (1.03–3.10)	2.71 (1.51–3.65)	0.06	1.91 (1.20–3.35)	3.43 (1.84–5.40)	<0.01	
Clinical outcomes	
Length of stay (days)	14.00
(8.00–22.00)	24.50
(10.75–46.50)	<0.01	10.00
(5.00–18.00)	24.00
(16.00–42.00)	<0.01	10.00
(6.00–16.00)	24.00
(15.00–48.25)	<0.01	9.00
(5.00–16.00)	21.00
(15.50–35.00)	<0.01	
Discharge within 10 days	145 (29.71)	5 (6.1)	<0.01	912 (44.38)	4 (1.42)	<0.01	444 (49.22)	5 (3.16)	<0.01	461 (53.42)	5 (4.85)	<0.01	
ICU admission	33 (6.76)	82 (100.00)	<0.01	307 (14.94)	281 (100.00)	<0.01	124 (13.69)	158 (100)	<0.01	90 (10.43)	103 (100)	<0.01	
In-hospital death	55 (11.27)	30 (36.59)	<0.01	303 (14.74)	145 (51.6)	<0.01	62 (6.84)	71 (44.94)	<0.01	58 (6.72)	38 (36.89)	<0.01	
Values shown are the median (IQR) for quantitative variables and n (%) for categorical variables.
Quantitative variables were compared through T-tests or Mann-Whitney U test. according to their distributions. Categorical variables were compared by Chi-squared test. Significance level ≤0.05.


Supplementary Table 4. Admission to ICU in the four pandemic waves
	Wave 1 (n=570)	Wave 2 (n=2336)	Wave 3 (n=1064)	Wave 4 (n=966)	
	

No ICU	

ICU	p-value	

No ICU	

ICU	p-value	

No ICU	

ICU	p-value	

No ICU	

ICU	p-value	
Demographics	
No. patients	455	115		1748	588		782	282		773	193		
Age (years)	68 (53–80)	72 (60–79)	0.07	69 (54–81)	68 (59–76)	0.08	62 (48–75)	66 (56–75)	0.03	63 (47–77)	66 (55–75)	0.43	
Male	260 (57.14)	80 (69.57)	0.02	851 (48.68)	418 (71.09)	<0.01	412 (52.69)	188 (66.67)	<0.01	377 (48.77)	121 (62.69)	<0.01	
BMI (kg/m2)	25.20
(23.05–27.34)	26.92
(24.45–29.41)	<0.01	25.71
(23.44–28.72)	27.32
(24.74–30.70)	<0.01	26.23
(23.88–29.38)	28.06
(24.89–33.10)	<0.01	26.12
(22.99–29.56)	27.68
(25.32–31.05)	<0.01	
Comorbidities	
Obesity
(BMI >30 kg/m2)	55 (12.73)	17 (24.29)	0.02	274 (19.26)	95 (28.79)	<0.01	170 (23.22)	76 (35.85)	<0.01	148 (24.54)	45 (34.09)	0.03	
Diabetes	62 (13.66)	16 (13.91)	1	308 (17.91)	119 (20.24)	0.22	94 (12.18)	46 (16.31)	0.08	111 (14.36)	44 (22.8)	0.01	
Hypertension	148 (32.6)	54 (46.96)	<0.01	671 (39.01)	293 (49.83)	<0.01	264 (34.2)	114 (40.43)	0.07	255 (32.99)	79 (40.93)	0.04	
Malignancy	52 (11.45)	10 (8.7)	0.5	227 (13.2)	62 (10.54)	0.1	74 (9.59)	20 (7.09)	0.22	124 (16.04)	26 (13.47)	0.44	
Neurological disease	35 (7.71)	5 (4.35)	0.31	133 (7.73)	14 (2.38)	<0.01	30 (3.89)	5 (1.77)	0.12	40 (5.17)	5 (2.59)	0.18	
Stroke	15 (3.3)	6 (5.22)	0.4	93 (5.32)	37 (6.29)	0.41	24 (3.07)	9 (3.19)	1	19 (2.46)	6 (3.11)	0.61	
Cardiovascular disease	25 (5.51)	8 (6.96)	0.51	128 (7.44)	54 (9.18)	0.18	45 (5.83)	17 (6.03)	0.88	51 (6.6)	13 (6.74)	1	
Pneumopathy	55 (12.11)	17 (14.78)	0.43	165 (9.59)	76 (12.93)	0.03	58 (7.51)	29 (10.28)	0.16	68 (8.8)	17 (8.81)	1	
Gastrointestinal disease	10 (2.2)	2 (1.74)	1	35 (2.03)	8 (1.36)	0.38	17 (2.2)	6 (2.13)	1	24 (3.1)	6 (3.11)	1	
Chronic kidney disease	22 (4.85)	4 (3.48)	0.63	118 (6.86)	43 (7.31)	0.71	32 (4.15)	9 (3.19)	0.59	71 (9.18)	20 (10.36)	0.58	
Chronic liver disease	6 (1.32)	2 (1.74)	0.67	15 (0.87)	7 (1.19)	0.47	9 (1.17)	3 (1.06)	1	10 (1.29)	2 (1.04)	1	
Cirrhosis	0 (0)	1 (0.87)	0.2	19 (1.1)	6 (1.02)	1	7 (0.91)	0 (0)	0.2	17 (2.2)	5 (2.59)	0.79	
Thrombosis/ pulmonary embolism	12 (2.64)	11 (9.57)	<0.01	34 (1.95)	14 (2.38)	0.5	17 (2.17)	14 (4.96)	0.02	8 (1.03)	7 (3.63)	0.02	
Immunodeficiency	10 (2.2)	1 (0.87)	0.7	33 (1.92)	19 (3.23)	0.08	18 (2.33)	3 (1.06)	0.22	29 (3.75)	13 (6.74)	0.08	
Symptoms at admission	
Fever	211 (77.86)	46 (73.02)	0.41	595 (59.03)	192 (59.44)	0.95	404 (69.06)	114 (57.29)	<0.01	257 (57.49)	52 (40.94)	<0.01	
Anosmia/ dysgeusia	13 (5.31)	2 (3.33)	0.74	58 (7.17)	5 (1.61)	<0.01	22 (4.21)	2 (0.94)	0.02	7 (1.91)	0 (0)	0.2	
Cough	134 (50.95)	29 (45.31)	0.49	281 (30.85)	91 (28.09)	0.36	189 (34.36)	42 (20.29)	<0.01	138 (34.16)	27 (21.26)	0.01	
Dyspnea	140 (52.43)	40 (61.54)	0.21	492 (51.09)	183 (59.8)	0.01	336 (59.47)	118 (59)	0.93	209 (48.6)	58 (45.31)	0.55	
Myalgia/arthralgia	44 (17.53)	14 (22.58)	0.36	161 (18.46)	47 (14.46)	0.12	128 (23.23)	24 (11.37)	<0.01	76 (19.24)	9 (6.92)	<0.01	
Gastrointestinal	36 (13.9)	7 (11.48)	0.83	76 (9.12)	22 (7.01)	0.29	48 (9.09)	9 (4.25)	0.03	29 (7.63)	1 (0.78)	<0.01	
Hospital admission data	
Oxygen saturation (%)	96 (94–98)	95 (93–97)	0.01	96 (94–97)	94 (92.5–96)	<0.01	96 (94.5–97.6)	95 (94–97)	<0.01	96 (95–98)	95.2 (93.2–96)	0.01	
Body temperature (°C)	37 (36–38)	37.8 (36.8–38.75)	<0.01	36.2 (36–37.1)	36.5 (36–37.8)	<0.01	36.3 (36–37.5)	36.4 (36–37.6)	0.31	36.3 (36–37.3)	36.4 (36–37.2)	0.75	
Cardiac frequency (bpm)	78 (70–88)	84 (77.5–98)	<0.01	80 (72–89)	79 (71–89)	0.83	80 (73–90)	80 (72.5–90)	0.97	80 (73–88)	80.5 (71.5–88)	0.69	
Respiratory (rate per minute)	13 (12–15)	14 (12–15)	0.14	13 (12–14)	13 (12–15)	0.05	14 (12–16)	14 (13–18)	<0.01	14 (13–15)	15 (13–17)	0.01	
Mean blood pressure (mmHg)	90. (83–97)	93 (85–100)	0.04	90 (83–99)	91 (83–100)	0.3	92 (84–100)	93 (85–103)	0.16	91 (83–101)	92 (86.–102)	0.63	
P/F	321.15
(213.47–379.28)	184.30
(123.73–318.98)	<0.01	242.85
(170.85–329.05)	134.00
(103.30–212.15)	<0.01	223.65
(161.12–319.00)	127.00
(101.40–168.65)	<0.01	211.75
(150.00–301.70)	125.00
(91.28–155.75)	<0.01	
Laboratory tests	
Hemoglobin (g/dl)	13.6
(12.10–14.80)	13.6
(12.15–14.88)	0.91	13
(11.40–14.30)	13.8
(11.90–14.97)	<0.01	13.3
(12.00–14.70)	13.65
(12.12–15.00)	0.14	13.1
(11.50–14.50)	13
(11.50–14.60)	0.33	
Haematocrit (%)	40.20
(36.70–43.40)	40.60
(36.25–43.53)	0.81	38.50
(34.40–42.10)	40.50
(35.80–44.00)	<0.01	39.00
(35.60–42.90)	40.00
(36.00–43.88)	0.06	38.10
(33.70–41.20)	37.30
(33.02–42.00)	0.79	
Platelets (cell × 109/L)	209.00
(164.75–270.00)	199.00
(152.25–262.75)	0.29	222.00
(173.00–287.00)	210.00
(158.00–278.50)	<0.01	202.00
(159.25–265.75)	191.50
(149.00–259.00)	0.07	206.00
(155.00–268.00)	210.50
(155.00–280.75)	0.76	
WBC
(cell × 109/L)	6.11
(4.80–8.39)	7.70
(5.51–9.65)	<0.01	7.96
(5.78–11.05)	8.52
(6.07–11.40)	0.02	7.18
(5.17–9.74)	7.85
(5.77–10.92)	<0.01	7.26
(5.47–10.59)	8.62
(6.15–12.15)	0.01	
Neutrophils (cell × 109/L)	4.48
3.22–6.32)	5.92
(4.18–7.84)	<0.01	6.11
(4.10–9.07)	6.95
(4.72–9.45)	<0.01	5.44
(3.71–7.70)	6.53
(4.31–9.37)	<0.01	5.49
(3.82–8.26)	6.98
(4.56–10.68)	<0.01	
Lymphocytes (cell × 109/L)	1.14
(0.82–1.51)	0.93
(0.68–1.27)	<0.01	1.13
(0.80–1.60)	0.90
(0.62–1.36)	<0.01	1.01
(0.73–1.46)	0.79
(0.58–1.15)	<0.01	1.07
(0.76–1.58)	0.80
0.59–1.20)	<0.01	
D-dimer (ng/mL)	907.00
(480.50–1781.50)	1489.00
(732.00–2998.00)	<0.01	900.00
(472.50–2002.00)	980.50
(531.00–2260.00)	0.13	711.00
(402.00–1412.00)	876.00
(518.00–2253.50)	<0.01	848.00
(501.00–1903.50)	1503.50
(714.25–4174.75)	<0.01	
Procalcitonin (ng/mL)	0.12
(0.07–0.26)	0.13
(0.08–0.28)	0.26	0.12
(0.07–0.29)	0.16
(0.09–0.39)	<0.01	0.09
(0.07–0.18)	0.14
(0.08–0.30)	<0.01	0.13
(0.08–0.36)	0.20
(0.09–0.67)	0.25	
IL-6 (ng/L)	14.60
7.80–29.70)	24.15
(14.28–70.78)	0.01	19.50
(8.60–40.70)	24.00
(10.05–53.65)	0.04	18.60
(8.10–35.70)	27.90
(11.60–64.50)	<0.01	23.60
(10.50–52.03)	35.10
(14.25–74.35)	0.01	
HS troponin (ng/L)	10.00
(5.00–32.25)	21.50
(12.25–65.25)	<0.01	13.00
(6.00–33.00)	17.00
(7.00–76.00)	<0.01	8.50
(5.00–21.00)	16.50
(8.00–51.75)	<0.01	11.50
(5.00–31.25)	27.00
(11.00–99.00)	0.04	
Lactate (mmol/L)	1.20
(0.80–1.60)	1.00
(0.78–1.77)	0.82	1.10
(0.80–1.60)	1.20
(0.90–1.50)	0.16	1.00
(0.70–1.40)	1.10
(0.80–1.70)	0.01	1.00
(0.70–1.30)	1.10
(0.90–1.50)	0.19	
Lactic dehydrogenase (IU/L)	277.00
(214.00–390.25)	382.00
(294.50–610.00)	<0.01	289.00
(219.00–395.00)	378.00
(276.00–530.00)	<0.01	309.00
(229.75–422.00)	395.50
(293.00–545.50)	<0.01	292.00
(213.00–400.00)	374.00
(266.00–546.00)	<0.01	
Glucose (mg/dL)	107.00
95.00–124.00)	118.00
(103.50–140.00)	<0.01	112.00
(94.00–138.00)	130.00
(109.00–169.00)	<0.01	111.00
(95.00–131.75)	128.00
(109.00–161.75)	<0.01	109.00
(95.00–130.00)	141.50
(117.25–202.50)	<0.01	
Creatinine (mg/dL)	0.88
(0.71–1.10)	0.96
(0.78–1.28)	<0.01	0.86
(0.68–1.15)	0.94
(0.74–1.25)	<0.01	0.82
(0.66–1.01)	0.85
(0.67–1.09)	0.07	0.89
(0.70–1.17)	0.92
(0.70–1.26)	0.03	
ALT (IU/L)	22.50
(15.00–37.00)	31.50
(17.25–46.75)	<0.01	21.00
(13.00–35.00)	29.00
(18.00–46.50)	<0.01	26.00
(16.00–43.00)	31.00
(19.75–45.00)	<0.01	21.00
(13.00–37.00)	28.00
(17.00–48.00)	<0.01	
AST (IU/L)	30.00
(22.00–47.50)	46.00
(33.50–60.50)	<0.01	29.00
(20.00–45.00)	34.50
(24.00–61.50)	0.01	31.00
(21.00–45.00)	44.00
(25.75–74.50)	<0.01	30.00
(21.25–46.00)	36.50
(23.25–60.25)	<0.01	
GGT (IU/L)	30.50
(18.00–58.25)	46.00
(23.00–61.00)	0.03	30.00
(19.00–58.00)	41.00
(24.00–71.00)	<0.01	34.00
(21.00–58.00)	42.00
(25.00–81.00)	<0.01	30.00
(19.00–60.00)	35.00
(22.00–67.00)	0.42	
ALP (IU/L)	70.00
(55.75–89.00)	59.00
(49.00–79.00)	0.02	68.00
(54.00–90.00)	61.00
(49.00–80.00)	<0.01	60.00
(48.00–81.00)	56.00
(45.75–76.00)	0.06	67.00
(52.00–97.00)	62.00
(52.00–97.00)	0.07	
Total bilirubin (mg/dL)	0.60
(0.40–0.80)	0.60
(0.40–0.90)	0.21	0.60
(0.40–0.80)	0.60
(0.50–0.80)	0.03	0.60
(0.50–0.80)	0.60
(0.40–0.80)	0.83	0.60
(0.40–0.80)	0.60
(0.50–0.88)	0.04	
Direct bilirubin (mg/dL)	0.50
(0.30–0.50)	0.55
(0.30–1.22)	0.61	0.60
(0.50–0.90)	0.65
(0.50–1.05)	0.83	0.50
(0.40–0.95)	0.60
(0.50–0.90)	0.39	0.60
(0.40–1.70)	1.00
(0.50–2.25)	0.08	
Albumin (g/L)	35.00
(31.00–38.00)	30.00
(27.00–34.00)	<0.01	32.00
(28.25–35.00)	30.00
(27.00–34.00)	<0.01	33.00
(30.00–36.00)	31.00
(28.00–33.00)	<0.01	33.00
(29.00–36.00)	29.00
(25.00–32.00)	<0.01	
Total cholesterol (mg/dL)	136.00
(115.00–170.50)	123.00
(99.00–150.00)	<0.01	145.00
(120.75–169.25)	133.00
(113.00–157.75)	<0.01	140.00
(118.00–167.00)	130.00
(113.50–154.50)	0.01	137.50
(113.00–165.00)	132.00
(108.00–154.00)	0.01	
HDL cholesterol (mg/dL)	31.00
(26.00–41.00)	24.00
(20.00–31.00)	<0.01	35.00
(28.00–43.00)	32.00
(24.00–39.00)	<0.01	33.00
(26.00–40.25)	32.00
(27.00–39.50)	0.36	36.00
(28.00–43.00)	28.00
(22.00–38.00)	<0.01	
Triglycerides (mg/dL)	108.50
(88.00–150.00)	130.00
(94.00–183.00)	0.12	118.00
(92.00–155.00)	127.00
(98.00–173.50)	0.01	115.00
(89.00–149.00)	131.50
(102.00–169.50)	<0.01	108.00
(85.00–147.75)	139.50
(99.50–189.50)	<0.01	
INR	1.04
(1.00–1.09)	1.07
(1.02–1.13)	<0.01	1.03 (0.98–1.09)	1.04
(0.99–1.13)	<0.01	1.07
(1.02–1.13)	1.08
(1.03–1.14)	0.03	1.02
(0.97–1.08)	1.06
(1.00–1.12)	<0.01	
Noninvasive tests	
FIB-4	1.85
(1.17–3.10)	2.45
(1.78–4.43)	0	1.72
(0.89–3.07)	2.09
(1.31–3.72)	0.01	1.82
(0.97–2.79)	2.71
(1.41–4.45)	0.01	1.90
(1.19–3.32)	2.71
(1.61–5.39)	0	
Clinical outcomes	
Length
of stay (days)	13.00
(8.00–20.00)	25.00
(14.00–42.00)	<0.01	9.00
(5.00–16.00)	20.00
(13.00–31.25)	<0.01	9.00
(5.00–15.00)	20.00
(13.00–35.00)	<0.01	9.00
5.00–15.00)	18.00
(13.00–31.00)	<0.01	
Discharge within 10 days	145 (31.87)	5 (4.35)	<0.01	873 (49.94)	43 (7.31)	<0.01	432 (55.53)	17 (6.03)	<0.01	447 (57.83)	19 (9.84)	<0.01	
Mechanical ventilation	0 (0)	82 (71.3)	<0.01	0 (0.00)	281 (47.79)	<0.01	0 (0.00)	158 (56.03)	<0.01	0 (0.00)	103 (53.37)	<0.01	
In-hospital death	48 (10.55)	37 (32.17)	<0.01	249 (14.24)	199 (33.84)	<0.01	52 (6.65)	81 (28.72)	<0.01	45 (5.82)	51 (26.42)	<0.01	
Values shown are the median (IQR) for quantitative variables and number (%) for categorical variables.
Quantitative variables were compared through T-tests or Mann-Whitney U test. according to their distributions. Categorical variables were compared by Chi-squared test. Significance level ≤0.05.
Supplementary Table 5. Discharge in 10 days
	Wave 1 (n=570)	Wave 2 (n=2336)	Wave 3 (n=1060)	Wave 4 (n=966)	
	

Discharge in 10 days	

No discharge in 10 days	p-value	

Discharge in 10 days	

No discharge in 10 days	p-value	

Discharge n 10 days	

No discharge in 10 days	p-value	

Discharge in 10 days	

No discharge in 10 days	p-value	
Demographics	
No. patients	150	420		916	1420		453	611		466	500		
Age (years)	57 (48–69)	72 (58–82)	<0.01	59 (46–73)	74 (62–82)	<0.01	55 (40–69)	69 (57–77)	<0.01	57.5 (39–73)	69 (57–79)	<0.01	
Male	84 (56)	256 (60.95)	0.29	418 (45.63)	851 (59.93)	<0.01	231 (50.99)	368 (60.23)	<0.01	213 (45.71)	285 (57)	<0.01	
BMI (kg/m2)	25.20
(23.44–27.01)	25.39
(23.23–27.76)	0.37	25.95
(23.83–29.05)	25.95
(23.62–29.30)	0.66	26.30
(23.67–29.90)	26.89
(24.22–30.86)	0.07	26.23
(22.86–30.10)	26.73
(23.88–30.16)	0.2	
Comorbidities	
Obesity
(BMI >30 kg/m2)	14 (10)	58 (16.02)	0.09	151 (20.94)	218 (21.12)	0.95	102 (22.52)	144 (27.12)	0.41	89 (25.65)	104 (26.8)	0.74	
Diabetes	12 (8)	66 (15.75)	0.02	134 (14.74)	293 (20.94)	<0.01	34 (7.51)	106 (17.46)	<0.01	49 (10.52)	106 (21.2)	<0.01	
Hypertension	29 (19.33)	173 (41.29)	<0.01	295 (32.45)	669 (47.82)	<0.01	113 (24.94)	265 (43.66)	<0.01	128 (27.47)	206 (41.2)	<0.01	
Malignancy	6 (4)	56 (13.37)	<0.01	95 (10.45)	194 (13.87)	0.02	30 (6.62)	64 (10.54)	0.04	73 (15.67)	77 (15.4)	0.93	
Neurological disease	4 (2.67)	36 (8.59)	0.01	27 (2.97)	120 (8.58)	<0.01	9 (1.99)	26 (4.28)	0.05	17 (3.65)	28 (5.6)	0.17	
Stroke	1 (0.67)	20 (4.76)	0.02	23 (2.51)	107 (7.54)	<0.01	8 (1.77)	25 (4.09)	0.03	10 (2.15)	15 (3)	0.43	
Cardiovascular disease	2 (1.33)	31 (7.4)	<0.01	35 (3.85)	147 (10.51)	<0.01	10 (2.21)	52 (8.57)	<0.01	26 (5.58)	38 (7.6)	0.24	
Pneumopathy	7 (4.67)	65 (15.51)	<0.01	45 (4.95)	196 (14.01)	<0.01	30 (6.62)	57 (9.39)	0.14	28 (6.01)	57 (11.4)	<0.01	
Gastrointestinal disease	1 (0.67)	11 (2.63)	0.2	12 (1.32)	31 (2.22)	0.16	6 (1.32)	17 (2.8)	0.14	7 (1.5)	23 (4.6)	0.01	
Chronic kidney disease	3 (2)	23 (5.49)	0.11	25 (2.75)	136 (9.72)	<0.01	9 (1.99)	32 (5.27)	0.01	27 (5.79)	64 (12.8)	<0.01	
Chronic liver disease	0 (0)	8 (1.91)	0.12	5 (0.55)	17 (1.22)	0.13	5 (1.10)	7 (1.15)	1	5 (1.07)	7 (1.4)	0.77	
Cirrhosis	0 (0)	1 (0.24)	1	5 (0.55)	20 (1.43)	0.06	2 (0.44)	5 (0.82)	0.71	7 (1.5)	15 (3)	0.13	
Thrombosis/pulmonary embolism	0 (0)	23 (5.48)	<0.01	9 (0.98)	39 (2.75)	<0.01	7 (1.55)	24 (3.93)	0.03	1 (0.21)	14 (2.8)	<0.01	
Immunodeficiency	3 (2)	8 (1.91)	1	12 (1.32)	40 (2.86)	0.01	6 (1.32)	15 (2.47)	0.27	14 (3)	28 (5.6)	0.06	
Symptoms at admission	
Fever	58 (72.5)	199 (78.35)	0.29	316 (52.75)	471 (64.34)	<0.01	223 (49.23)	295 (67.51)	0.36	126 (44.52)	183 (62.89)	<0.01	
Anosmia/ dysgeusia	6 (8.7)	9 (3.81)	0.11	40 (8.06)	23 (3.69)	<0.01	16 (3.53)	8 (1.9)	0.02	2 (0.83)	5 (1.96)	0.45	
Cough	44 (57.89)	119 (47.41)	0.12	169 (30.51)	203 (29.81)	0.8	118 (26.05)	113 (26.28)	<0.01	73 (28.08)	92 (33.95)	0.16	
Dyspnea	40 (51.28)	140 (55.12)	0.6	228 (41.08)	447 (62.61)	<0.01	179 (39.51)	275 (63.95)	<0.01	99 (36.13)	168 (59.15)	<0.01	
Myalgia/arthralgia	12 (17.39)	46 (18.85)	0.86	91 (17.27)	117 (17.46)	0.94	71 (15.67)	81 (18.62)	0.31	41 (15.89)	44 (16.48)	0.91	
Gastrointestinal	16 (21.05)	27 (11.07)	0.03	43 (8.43)	55 (8.63)	0.92	32 (7.06)	25 (5.88)	0.04	15 (6)	15 (5.79)	1	
Hospital admission data	
Oxygen saturation (%)	97 (95–98)	96 (94–97)	0.01	96 (94.5–97.)	95 (93–97)	<0.01	96 (95–98)	95 (94–97)	<0.01	96.8 (95–98)	96 (94–97)	<0.01	
Body emperature (°C)	37.2 (36.–38)	37.1 (36.2–38)	0.72	36.2 (36–37)	36.3 (36–37.)	<0.01	36.2 (36–37.4)	36.3 (36–37.5)	0.42	36.3 (36–37)	36.4 (36–37.4)	0.36	
Cardiac frequency (bpm)	7 (70–87.5)	80 (71–90)	0.04	80 (72–88)	80 (72–90)	0.03	80 (72–88.5)	80 (73–91)	0.12	80 (72–85)	81 (74–90)	0.02	
Respiratory (rate per minute)	12 (12–14)	14 (12–15)	<0.01	13 (12–14)	13 (12–15)	0.03	14 (12–15)	14 (13–17.5)	<0.01	14 (12–15)	15 (13–16)	<0.01	
Mean blood pressure (mmHg)	88 (82–97)	90 (83–97)	0.1	90 (83–97.5)	90 (83–100)	0.79	92 (83–100)	93 (85–102)	0.14	90 (83–100)	93 (86–103)	<0.01	
P/F	353.15
(288.75–401.00)	278.15
(178.07–361.90)	<0.01	267.20
(206.88–345.82)	185.70
(123.00–279.00)	<0.01	257.70
(195.77–333.42)	164.30
(115.80–247.30)	<0.01	234.65
(176.95–328.70)	166.60
(122.30–244.95)	<0.01	
Laboratory tests	
Hemoglobin (g/dL)	14.10
(12.90–14.88)	13.50
(11.80–14.80)	<0.01	13.30
(11.90–14.60)	13.10
(11.20–14.40)	<0.01	13.60
(12.40–14.97)	13.30
(11.70–14.70)	0.01	13.20
(11.50–14.50)	13.00
(11.50–14.50)	0.37	
Haematocrit (%)	40.90
(37.90–43.27)	40.00
(35.88–43.42)	0.04	39.10
(35.30–42.60)	38.80
(33.90–42.70)	0.02	39.80
(36.50–43.70)	38.90
(35.30–42.90)	0.01	38.20
(33.80–41.27)	37.40
(33.50–41.30)	0.4	
Platelets (cell × 109/L)	204.50
(163.50–264.75)	209.50
(162.75–267.75)	0.64	227.00
(180.00–286.00)	212.00
(160.50–282.50)	<0.01	204.00
(161.00–258.00)	196.00
(150.00–267.00)	0.12	215.00
(163.00–271.00)	197.00
(148.75–270.00)	0.02	
WBC (cell × 109/L)	6.20 (4.94–7.97)	6.46 (4.87–9.07)	0.12	7.93 (5.88–10.77)	8.25 (5.83–11.44)	0.18	7.15 (5.31–9.55)	7.52 (5.19–10.56)	0.18	7.41 (5.60–10.55)	7.76 (5.56–11.16)	0.43	
Neutrophils (cell × 109/L)	4.42 (3.21–6.14)	4.89 (3.51–7.04)	0.03	5.93 (4.14–8.59)	6.56 (4.33–9.46)	<0.01	5.40 (3.82–7.67)	5.93 (3.96–8.64)	0.03	5.52 (3.92–8.24)	5.83 (4.05–9.11)	0.04	
Lymphocytes (cell × 109/L)	1.23 (0.89–1.57)	1.05 (0.79–1.47)	0.01	1.21 (0.86–1.76)	1.00 (0.67–1.41)	<0.01	1.05 (0.76–1.45)	0.88 (0.64–1.26)	<0.01	1.12 (0.78–1.62)	0.94 (0.65–1.40)	<0.01	
D-dimer (ng/mL)	660.00
(409.00–1065.50)	1093.00
(606.00–2410.00)	<0.01	771.00
(408.00–1657.25)	1031.50
(548.25–2365.75)	<0.01	635.00
(382.00–1134.00)	872.00
(483.00–1970.00)	<0.01	834.00
(453.00–2011.00)	1123.00
(598.25–3057.75)	<0.01	
Procalcitonin (ng/mL)	0.10 (0.06–0.18)	0.13 (0.08–0.31)	0.02	0.09 (0.06–0.18)	0.15 (0.08–0.40)	<0.01	0.09 (0.06–0.13)	0.13 (0.07–0.29)	<0.01	0.11 (0.07–0.22)	0.18 (0.09–0.52)	<0.01	
IL-6 (ng/L)	6.90
(4.68–15.50)	18.00
(9.30–36.15)	<0.01	15.50
(6.90–31.95)	24.10
(10.20–53.70)	<0.01	16.80
(7.60–32.80)	24.50
(10.00–54.12)	<0.01	19.00
(9.40–41.05)	29.10
(13.55–70.73)	<0.01	
HS troponin (ng/L)	7.00
(4.00–14.75)	17.00
(8.75–47.00)	<0.01	9.00
(5.00–22.00)	18.00
(7.25–57.00)	<0.01	7.00
(4.00–12.00)	15.00
6.00–32.50)	<0.01	11.00
(5.00–23.00)	17.00
(7.00–61.75)	<0.01	
Lactate (mmol/L)	1.00 (0.85–1.30)	1.20 (0.80–1.70)	0.6	1.00 (0.70–1.40)	1.20 (0.90–1.70)	<0.01	0.90 (0.70–1.30)	1.00 (0.80–1.60)	<0.01	1.00 (0.70–1.30)	1.00 (0.70–1.40)	0.1	
Lactic dehydrogenase (IU/L)	256.00
(206.00–339.00)	321.00
(234.75–438.00)	<0.01	270.00
(210.25–354.75)	338.00
(243.00–473.00)	<0.01	295.00
(223.00–410.00)	361.00
(258.00–477.00)	<0.01	271.00
(196.75–388.25)	333.50
(253.00–465.75)	<0.01	
Glucose (mg/dL)	103.00
(93.50–116.00)	112.00
(97.00–134.00)	<0.01	110.00
(92.00–130.00)	121.00
(101.00–157.00)	<0.01	107.00
(92.00–129.00)	122.00
(102.75–151.00)	<0.01	106.00
(93.00–126.00)	121.00
(103.00–158.00)	<0.01	
Creatinine (mg/dL)	0.86 (0.70–1.02)	0.92 (0.74–1.21)	0.01	0.79 (0.66–0.99)	0.94 (0.73–1.31)	<0.01	0.79 (0.64–0.96)	0.85 (0.69–1.12)	<0.01	0.82 (0.67–1.05)	0.97 (0.74–1.36)	<0.01	
ALT (IU/L)	25.00
(16.00–40.00)	24.00
(15.00–38.00)	0.99	22.00
(14.00–37.00)	23.50
(14.00–39.00)	0.37	27.00
(17.00–48.00)	27.00
(18.00–42.00)	0.91	21.00
(13.00–37.00)	23.00
(14.00–39.00)	0.08	
AST (IU/L)	29.50
(23.50–41.75)	35.00
(24.00–56.00)	0.12	26.00
(18.00–38.25)	35.00
(23.00–54.25)	<0.01	29.00
(20.00–43.50)	36.00
(23.00–56.75)	0.05	29.50
(21.00–46.25)	32.00
(22.00–49.00)	0.11	
GGT (IU/L)	31.00
(18.00–56.50)	35.00
(19.50–61.00)	0.32	32.00
(19.00–57.75)	35.00
(20.00–65.00)	0.05	34.00
(21.00–58.00)	36.00
(23.00–67.00)	0.23	29.00
(18.00–57.25)	34.00
(21.00–64.75)	0.01	
ALP (IU/L)	63.50
(51.75–76.25)	71.00
(53.00–90.75)	0.04	66.00
(52.00–87.25)	66.00
(51.75–88.00)	0.87	60.00
(48.00–75.00)	58.00
(47.00–81.50)	0.99	65.00
(52.50–95.50)	64.00
(52.00–98.50)	1	
Total bilirubin (mg/dL)	0.60 (0.50–0.88)	0.60 (0.40–0.80)	0.19	0.60 (0.50–0.80)	0.60 (0.40–0.90)	0.89	0.60 (0.50–0.80)	0.60 (0.40–0.80)	0.59	0.60 (0.50–0.80)	0.60 (0.40–0.80)	0.67	
Direct bilirubin (mg/dL)	0.50 (0.30–0.50)	0.50 (0.30–1.12)	0.74	0.50 (0.45–0.80)	0.70 (0.50–1.12)	0.02	0.40 (0.40–0.55)	0.70 (0.50–1.10)	<0.01	0.55 (0.40–1.08)	0.80 (0.50–2.40)	0.1	
Albumin (g/L)	37.00
(34.00–41.00)	33.00
(29.00–37.00)	<0.01	33.00
(30.00–36.00)	30.00
(27.00–33.00)	<0.01	34.00
(30.00–37.00)	31.00
(29.00–34.00)	<0.01	33.00
(29.00–37.00)	31.00
(27.00–34.00)	<0.01	
Total cholesterol (mg/dL)	149.00
(126.00–174.00)	128.00
(107.50–159.50)	<0.01	149.00
(126.00–172.00)	136.00
(113.00–161.75)	<0.01	142.00
(120.00–170.25)	134.00
(114.00–160.00)	0.01	148.00
(123.50–169.00)	130.00
(108.25–154.00)	<0.01	
HDL cholesterol (mg/dL)	31.00
(27.00–40.25)	30.00
(23.00–40.00)	0.42	36.00
(29.00–43.00)	32.00
(25.00–41.00)	<0.01	33.00
(27.00–40.00)	32.00
(26.00–40.00)	0.2	37.00
(29.00–43.00)	32.00
(25.00–42.00)	<0.01	
Triglycerides (mg/dL)	110.00
(85.00–139.25)	110.00
(89.50–162.00)	0.24	118.00
(92.00–153.00)	122.00
(93.00–163.00)	0.18	115.00
(88.00–143.25)	122.00
(95.00–161.00)	0.01	107.00
(85.00–146.50)	117.00
(88.00–160.00)	0.04	
INR	1.03 (0.99–1.07)	1.06 (1.01–1.11)	<0.01	1.01 (0.96–1.07)	1.04 (0.99–1.13)	<0.01	1.05 (1.01–1.10)	1.08 (1.03–1.15)	<0.01	1.01 (0.96–1.06)	1.03 (0.98–1.13)	<0.01	
Noninvasive tests	
FIB-4	1.38 (1.07–2.08)	2.16 (1.33–3.72)	<0.01	1.36 (0.78–2.19)	2.29 (1.30–4.01)	<0.01	1.62 (0.79–2.45)	2.11 (1.35–3.66)	<0.01	1.71 (0.98–2.84)	2.43 (1.46–4.17)	<0.01	
Clinical outcomes	
Length of stay (days)	7.00
(5.00–8.75)	18.50
(13.00–30.00)	<0.01	6.00
(4.00–8.00)	18.00
(12.00–27.00)	<0.01	6.00
(4.00–8.00)	17.00
(13.00–28.00)	<0.01	5.00
(3.00–8.00)	18.00
(13.00–26.00)	<0.01	
ICU admission	5 (3.33)	110 (26.19)	<0.01	43 (4.69)	545 (38.38)	<0.01	17 (3.75)	265 (43.37)	<0.01	19 (4.08)	174 (34.8)	<0.01	
Mechanical ventilation	5 (3.33)	77 (18.33)	<0.01	4 (0.44)	277 (19.51)	<0.01	5 (1.10)	153 (25.04)	<0.01	5 (1.07)	98 (19.6)	<0.01	
In-hospital death	0 (0.00)	85 (20.24)	<0.01	0 (0)	448 (31.55)	<0.01	0 (0.00)	133 (21.77)	<0.01	0 (0.00)	96 (19.2)	<0.01	
Values shown are the median (IQR) for quantitative variables and number (percentage) for categorical variables.
Quantitative variables were compared through T-tests or Mann-Whitney U test. according to their distributions. Categorical variables were compared by Chi-squared test. Significance level ≤0.05.
